# Human endoderm stem cells reverse inflammation-related acute liver failure through cystatin SN-mediated inhibition of interferon signaling

**DOI:** 10.1038/s41422-022-00760-5

**Published:** 2023-01-20

**Authors:** Yilin Xu, Jinglin Wang, Haozhen Ren, Hao Dai, Ying Zhou, Xiongzhao Ren, Yang Wang, Sisi Feng, Xiaogang Deng, Jiaying Wu, Tianlong Fu, Tengfei Nie, Haifeng He, Tongkun Wei, Bing Zhu, Lijian Hui, Bin Li, Jing Wang, Hongyan Wang, Luonan Chen, Xiaolei Shi, Xin Cheng

**Affiliations:** 1 State Key Laboratory of Cell Biology, Shanghai Institute of Biochemistry and Cell Biology, Center for Excellence in Molecular Cell Science, Chinese Academy of Sciences, University of Chinese Academy of Sciences, Shanghai, China; 2 Department of Hepatobiliary Surgery, the Affiliated Drum Tower Hospital of Nanjing University Medical School, Nanjing, Jiangsu China; 3 Hepatobiliary Institute Nanjing University, Nanjing, Jiangsu China; 4 Institute of Brain-Intelligence Technology, Zhangjiang Laboratory, Shanghai, China; 5 Department of Immunology and Microbiology, Shanghai Institute of Immunology, Shanghai Jiao Tong University School of Medicine, Shanghai Jiao Tong University, Shanghai, China; 6 National Laboratory of Biomacromolecules, CAS Center for Excellence in Biomacromolecules, Institute of Biophysics, Chinese Academy of Sciences, Beijing, China; 7 Key Laboratory of Systems Biology, Hangzhou Institute for Advanced Study, University of Chinese Academy of Sciences, Chinese Academy of Sciences, Hangzhou, Zhejiang China

**Keywords:** Cell signalling, Multipotent stem cells, Innate immunity

## Abstract

Acute liver failure (ALF) is a life-threatening disease that occurs secondary to drug toxicity, infection or a devastating immune response. Orthotopic liver transplantation is an effective treatment but limited by the shortage of donor organs, the requirement for life-long immune suppression and surgical challenges. Stem cell transplantation is a promising alternative therapy for fulminant liver failure owing to the immunomodulatory abilities of stem cells. Here, we report that when transplanted into the liver, human endoderm stem cells (hEnSCs) that are germ layer-specific and nontumorigenic cells derived from pluripotent stem cells are able to effectively ameliorate hepatic injury in multiple rodent and swine drug-induced ALF models. We demonstrate that hEnSCs tune the local immune microenvironment by skewing macrophages/Kupffer cells towards an anti-inflammatory state and by reducing the infiltrating monocytes/macrophages and inflammatory T helper cells. Single-cell transcriptomic analyses of infiltrating and resident monocytes/macrophages isolated from animal livers revealed dramatic changes, including changes in gene expression that correlated with the change of activation states, and dynamic population heterogeneity among these cells after hEnSC transplantation. We further demonstrate that hEnSCs modulate the activation state of macrophages/Kupffer cells via cystatin SN (CST1)-mediated inhibition of interferon signaling and therefore highlight CST1 as a candidate therapeutic agent for diseases that involve over-activation of interferons. We propose that hEnSC transplantation represents a novel and powerful cell therapeutic treatment for ALF.

## Introduction

Acute liver failure (ALF) is a devastating liver disease characterized by the rapid development of a series of clinical manifestations and complications, including hepatic dysfunction, coagulopathy, encephalopathy, multiorgan failure, and ultimately death in half of cases.^1^ While viral infections and drug toxicity are the predominant causes of ALF in developing and developed countries, respectively, hypoxic hepatitis, neoplastic infiltration and metabolic liver diseases can also induce ALF; therefore, significant phenotypic heterogeneity has been observed. To date, the most effective treatment of ALF is orthotopic liver transplantation (OLT).^2^ Intraperitoneal transplantation of human primary hepatocytes has also been exploited in clinical trials as an alternative to OLT for treating ALF in children.^3^ Despite the significant benefits of these applications, they are limited due to the critical shortage of donor organs, the need for life-long immunosuppression and surgical/postoperative challenges.^1^ In addition, detoxification with extracorporeal artificial or bioartificial liver devices and high-volume plasma exchange have been developed as liver-supportive therapeutic approaches to bridge the gap between liver damage and restoration or OLT, although their clinical benefit for survival remains unclear.^4^ Therefore, the treatment of ALF calls for novel strategies.

A cure for ALF is expected to be achieved by improving local and systemic conditions for hepatic tissue repair and regeneration. It has been well recognized that the inflammatory response and its timely resolution play pivotal roles in liver regeneration after injury.^5^ Accumulating evidence has shown that tissue-resident macrophages (also known as Kupffer cells, which are derived from yolk sac hematopoietic progenitors)^6^ and infiltrating monocytes are key regulators of the inflammatory response, undergo dynamic phenotypic and functional changes in a time-dependent manner after liver injury; these cells cooperate with other innate and adaptive immune cells, hepatic sinusoidal endothelial cells, hepatic stellate cells and hepatocytes to orchestrate inflammation, resolution and tissue repair.^7^ Therefore, potential treatments aimed at alleviating or reversing ALF by modulating the systemic and local immune environments represent promising strategies.

Given the immunomodulatory function of mesenchymal stem cells (MSCs), the therapeutic potential of these cells for liver diseases has been investigated in multiple studies.^8^ It has been demonstrated that when transplanted, MSCs were able to alleviate liver injury and promote tissue regeneration by secreting trophic and immunomodulatory factors, including prostaglandin E2, indoleamine 2,3-dioxygenase (IDO), HGF, TGFβ, IL6 and IL10, in a systemic manner and by directly inhibiting T effector cells through the expression of PD-L1 and PD-L2.^9^ Additionally, differential roles of MSCs in the fibrogenesis and healing process have been observed after transplantation in the liver injury or restoration phases.^10^ Clinical trials have been carried out using autologous MSCs to treat patients with Mayo End-stage Liver Diseases.^11,12^ However, the application of MSC transplantation is undermined by unwanted myofibroblast differentiation potential and the susceptibility of these cells to spontaneous malignant transformation.^13,14^ Thus, it is of great interest to identify new stem cell types that are more suitable as therapeutic reagents for ALF treatment.

Human pluripotent stem cell (hPSC)-derived, self-renewable endoderm stem cells (hEnSCs, also known as endoderm progenitor cells/EP cells) are unique, as they are nontumorigenic in vivo, have robust hepatic differentiation capacity in vitro, and express a series of immunoregulatory factors.^15–17^ Therefore, hEnSCs may serve as a novel therapeutic stem cell source for liver diseases, including ALF.

Here, we use rodent and swine ALF models to evaluate the therapeutic effect of hEnSCs and demonstrate that when transplanted into the liver, these cells are able to effectively reverse hepatic injury and significantly improve the survival of the animals. We demonstrate that hEnSCs tune the local immune microenvironment by affecting both the innate and adaptive cell lineages. The transplantation of hEnSCs skews the activation state of liver macrophages (monocyte-derived infiltrating macrophages (MoMFs) and Kupffer cells) towards an anti-inflammatory state and reduces the numbers of the infiltrating monocytes/MoMFs and the inflammatory T helper cells in the liver. Single-cell transcriptomic analyses (scRNA-seq) performed on monocytes and macrophages isolated from animal livers reveal not only dramatic changes in gene expression that correlate with the activation state of these cells but also dynamic population heterogeneity among these cells after hEnSC transplantation. Finally, we demonstrate that hEnSCs might alleviate the inflammation-related liver injury via the suppression of interferon (IFN) signaling in MoMFs and Kupffer cells by a immunomodulatory protein cystatin SN (CST1) that interferes with the interaction of IFNs and their receptors by directly binding to interferon gamma receptor 1 (IFNGR1) and 2 (IFNGR2), and via the consequent activation of IL10 signaling. In conclusion, hEnSC transplantation represents a novel and powerful cell therapeutic treatment for ALF, and CST1 alone may serve as a candidate medication for diseases that involve over-activation of IFNs.

## Results

### hEnSC transplantation alleviates liver injury and improves survival in rodent and swine ALF models

hEnSCs were chosen as a candidate therapeutic reagent for the treatment of ALF for the following reasons: 1) they are nontumorigenic in vivo; 2) they possess extensive in vitro proliferation capacity, which supports mass production to meet the quantities required for curative effects; 3) hEnSC lines exhibit homogeneous transcriptomes at the single-cell level and are karyotypically stable during passaging;^16^ 4) human leukocyte antigens (HLAs) are not expressed in hEnSCs, suggesting low immunogenicity (Supplementary information, Fig. S1a); 5) they express a spectrum of immunomodulatory factors, including TGFβ1 and cystatins, which may tune the microenvironment of the liver (Supplementary information, Fig. S1a); and 6) they are endoderm specific and not able to generate fibrotic myofibroblasts. These unique features suggest that hEnSCs might serve as an appropriate reagent for stem cell therapy of ALF.

**Fig. 1:**
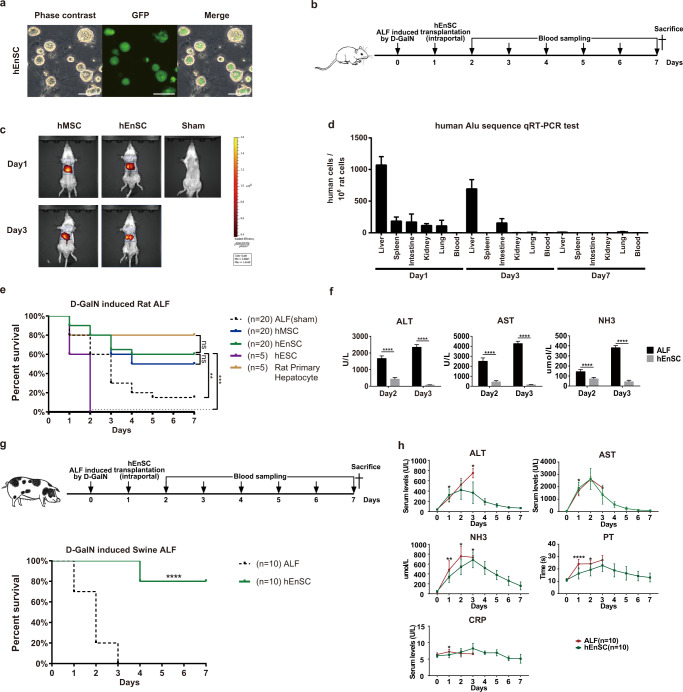
hEnSC transplantation alleviates liver injury and improves survival in both rodent and swine ALF models. **a** Morphology of EGFP-hEnSCs. Scale bars, 100 μm. **b** Schematic diagram of hEnSC transplantation in D-GalN-induced ALF rat model. **c** Live images showing in vivo distributions of transplanted DiR-labelled hEnSCs or hMSCs. **d** Quantification of human cells per 1 million rat cells by qRT-PCR designed to score the transcripts of human-specific *Alu* sequence across the rat tissues harvested on days 1, 3 and 7 post transplantation. **e** Kaplan–Meier survival curves of the rats transplanted with different cell types in D-GalN-induced ALF rat model. hMSC (*n* = 20), hEnSC (*n* = 20), rat primary hepatocyte (*n* = 5) and hESC (*n* = 5) were transplanted intraportally. hMSC, human mesenchymal stem cells; hEnSC, human endoderm stem cells; hESC, human embryonic stem cells. **f** Serum levels of ALT, AST and NH_3_ in ALF rats measured on day 2 and day 3 post drug administration. Blood samples were collected from 5 rats for each group. **g** Kaplan–Meier survival curves of PBS-treated ALF (*n* = 10) and hEnSC-transplanted ALF (*n* = 10) pigs in D-GalN-induced ALF model. **h** The dynamics of liver function and inflammation parameters measured in PBS-treated ALF (*n* = 10) and hEnSC-transplanted ALF (*n* = 10) pigs during 7 days post drug administration. NH_3_, blood ammonia; PT, prothrombin time; CRP, C-reactive protein; ns, no significance; *P < 0.05, **P < 0.01, ***P < 0.001, ****P < 0.0001. Log-rank (Mantel-Cox) test was used in **e** and **g**, Sidak’s multiple comparisons test in **f** and multiple *t*-tests in **h**. Error bars represent SD. See also Supplementary information, Figs. S1–S5 and Video S1.

To evaluate the efficacy of hEnSC transplantation for ALF treatment, we took advantage of two models of hepatotoxicity associated with ALF. First, the D-galactosamine hydrochloride (D-GalN)-induced and the D-GalN + lipopolysaccharide (D-GalN + LPS)-induced rat models, as well as the D-GalN-induced swine model were selected to mimick the inflammatory liver injury. D-GalN administration leads to panlobular hepatocyte apoptosis/necrosis and subsequent infiltration of polymorphonuclear cells by exhausting the uridine pool in hepatocytes and by exerting extrahepatic effects on gut permeability and endotoxemia.^18^ Simultaneous LPS treatment with D-GalN further sensitizes hepatocytes by activating Kupffer cells that secrete tumor necrosis factor-α (TNFα), leading to widespread apoptosis of hepatocytes.^19^ Second, the acetaminophen (APAP)-induced mouse model that represents the acute liver injury caused by direct damage of hepatocytes was also adopted.^20^ The efficacy of EGFP-hEnSCs (the hEnSC line that is derived from H9 human embryonic stem cell (hESC) line and carries lentiviral EGFP, Fig. 1a) in treating ALF was first compared with that of human MSCs, hESCs and primary rat hepatocytes in D-GalN-induced rat model. Briefly, 1 × 10^7^ cells were intraportally transplanted into the livers of wild-type rats 24 h post drug administration. Animal survival was monitored, and blood samples were collected every day until the time of death or sacrifice (Fig. 1b). As illustrated by fluorescence live imaging, the DiR-labeled hEnSCs kept homing to the host liver until day 3 (Fig. 1c), and became undetectable on day 7 post drug administration (data not shown). The quantitative real-time PCR (qRT-PCR) designed to detect the human specific *Alu* sequence in various tissues harvested from the transplanted animals revealed a predominant liver distribution of hEnSCs by day 3 and a clearance of these cells by day 7, which indicates that hEnSCs did not engraft and were eradicated in the wild-type rats shortly after transplantation (Fig. 1d), possibly either by Kupffer cells/MoMFs (as single-cell transcriptomic analysis and qRT-PCR revealed that hEnSCs do not express CD47) or by NK cells (as hEnSCs lack HLA expression) or by both (Supplementary information, Fig. S1a–c). This is in contrast with the fact that hEnSCs were able to engraft and differentiate into hepatocytes in immunocompromised Fah^–/–^Rag2^–/–^Il2rg^–/–^ (FRG) mice after intrasplenetic transplantation (Supplementary information, Fig. S1e). Approximately 90% of the ALF rats receiving intraportal injection of PBS buffer (sham-operated) died within 5 days, while 60% of hEnSC-transplanted animals survived 7 days before sacrifice (Fig. 1e; Supplementary information, Video S1). The survival rate of the hEnSC-transplanted group was comparable to those of the rat primary hepatocyte-transplanted group (80%) and the hMSC-transplanted group (40%) but was significantly better than that of the hESC-transplanted (0%) group (Fig. 1e). The levels of serum ALT, AST and blood ammonia (NH_3_) in the hEnSC-transplanted group were dramatically reduced compared to those of the sham-operated ALF group (Fig. 1f). H&E and TUNEL histochemical analyses revealed a significant reduction in inflammatory infiltration and cell death in the hEnSC-transplanted liver samples compared to the sham group (Supplementary information, Fig. S2a). In line with the aforementioned data, significant improvements with regard to liver morphology and animal behaviors were observed in the hEnSC-transplanted group relative to the sham group (Supplementary information, Fig. S2b and Video S1). However, differences in either pathology or hepatic injury parameters were not observed between the hEnSC- and the hMSC-transplanted groups (Supplementary information, Fig. S3a, b). Next, we used the D-GalN + LPS-induced ALF rat model that involves more severe inflammation to further test the efficacy of hEnSC transplantation. All the animals of the sham group died within 2 days; in contrast, 70% of the hEnSC-transplanted ALF rats survived. The survival rates of the groups transplanted with either rat primary hepatocytes (30%) or hMSCs (20%) were much lower than those of the hEnSC-transplanted groups (Supplementary information, Fig. S4a). Finally, hEnSCs derived from hESC line H1 (H1-hEnSCs) displayed similar efficacy to H9 hEnSCs in D-GalN-induced ALF rats, which indicates that the curative effect is not unique to specific cell line (Supplementary information, Fig. S4d–f). These data clearly demonstrated that hEnSC transplantation significantly alleviated hepatocyte injury in rodent ALF models.

The therapeutic effect of hEnSC transplantation was further investigated in D-GalN-induced ALF minipigs. Briefly, 5 × 10^8^ hEnSCs were harvested and intraportally transplanted into minipigs 24 h post D-GalN administration. Remarkably, 8 of 10 ALF minipigs transplanted with hEnSCs survived over 7 days before sacrifice, and the remaining two survived 4 days before death, while all of the sham-operated ALF animals died within 3 days (Fig. 1g). When compared to those of the PBS-treated ALF animals, the levels of serum ALT, AST, and blood NH_3_, prothrombin time (PT) and C-reactive protein (CRP) in the hEnSC-transplanted group were significantly reduced and rapidly returned to normal within a week (Fig. 1h), which is consistent with the reduced inflammatory infiltration and cell death in the hEnSC-transplanted livers as well as the improved behavioral phenotype of the transplanted pigs (Supplementary information, Fig. S5). These data indicated that hEnSC transplantation was able to protect porcine hepatocytes and rescue ALF pigs.

Finally, we also evaluated the effect of hEnSC transplantation in the APAP-induced ALF mouse model. More than 40% of the hEnSC-transplanted animals survived when 1 × 10^6^ hEnSCs were intraportally transplanted right after the administration of APAP (Supplementary information, Fig. S4b). No statistical significance in survival rates among the groups transplanted with either hEnSCs or hMSCs or mouse hepatocytes was observed. Overall, hEnSC transplantation displayed promising therapeutic potential in ameliorating ALF in both rodent and swine ALF models.

### hEnSC transplantation modulates the local immune system in ALF livers

Having established that hEnSC transplantation benefits the survival of ALF animals and protects hepatocytes from death, we next investigated the regulatory mechanism underpinning the alleviation of hepatic injury. Although hEnSCs can efficiently differentiate into hepatocytes and cholangiocytes both in vitro and in vivo,^15–17^ it is less likely that they differentiated into functional hepatic cells in vivo as they appeared to be cleared shortly after transplantation in the wild-type ALF animals (Fig. 1d). Since it has been widely accepted that the fine-tuned inflammatory response and its timely resolution are the central events that orchestrate tissue repair after injury,^5^ we speculated that transplanted hEnSCs may function by modulating the local immune microenvironment. Therefore, we investigated both the innate and adaptive immune responses upon hEnSC transplantation in D-GalN-induced ALF rat model.

**Fig. 2:**
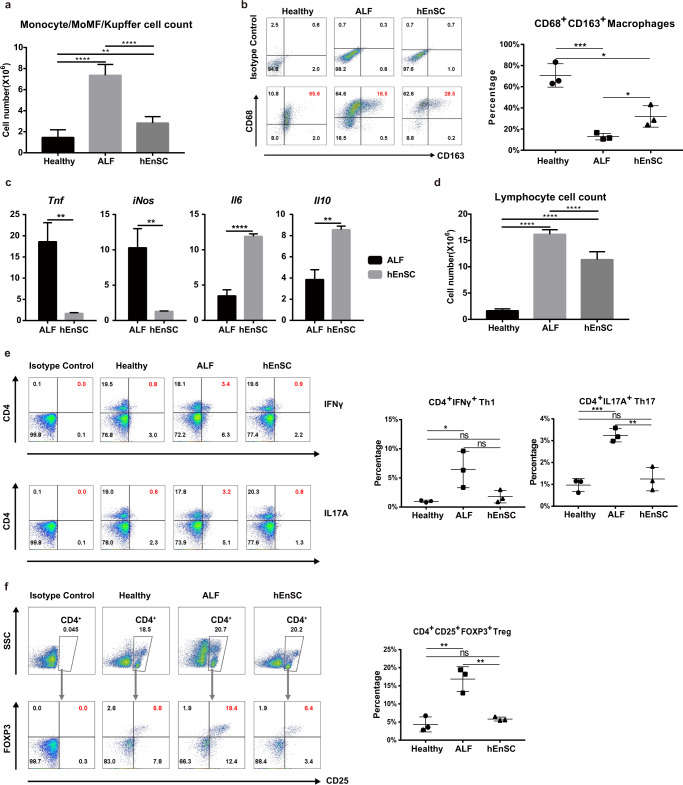
hEnSC transplantation modulates local immune system in ALF livers. **a** Cell counts of the monocyte/MoMF/Kupffer cell population isolated from rat livers 24 h post transplantation. **b** FACS analysis for CD68^+^CD163^+^ M2-like cells in the monocyte/MoMF/Kupffer cell populations isolated from ALF rat livers 24 h post transplantation; right panel: statistical differences among the groups calculated from the data of three independent experiments. **c** qRT-PCR showing expression of the indicated genes in the monocyte/MoMF/Kupffer cell samples isolated from PBS-treated ALF (ALF) and hEnSC-transplanted (hEnSC) rats; values were determined relative to *TATA-binding protein* (*Tbp*); *n* = 3 independent animals for ALF or hEnSC groups. **d** Cell counts of the lymphocytes isolated from rat livers 24 h post transplantation. **e** FACS analysis for CD4^+^IFNγ^+^ Th1 and CD4^+^IL17A^+^ Th17 cell populations in the lymphocytes isolated from ALF rat livers 24 h post transplantation; middle and right panels: statistical differences among the groups calculated from the data of three independent experiments on Th1 and Th17 populations. **f** FACS analysis for CD4^+^CD25^+^FOXP3^+^ Treg cells on the lymphocytes isolated from ALF rat livers 24 h post transplantation; right panel: statistical differences among the groups calculated from the data of three independent experiments. *P < 0.05, **P < 0.01, ***P < 0.001, ****P < 0.0001 (unpaired two-tailed Student’s *t*-tests). Error bars represent SD. See also Supplementary information, Figs. S6, S7.

We first examined infiltrating monocytes, MoMFs and Kupffer cells, as these cells are known to play pivotal regulatory roles during the initiation and resolution of tissue regeneration and are able to undergo drastic phenotypic and functional changes in a context-dependent manner.^7^ Compared to those in the healthy group, the total numbers of leukocytes harvested from the rat liver after perfusion increased by 403% and 93% in the sham and hEnSC-transplanted ALF groups, respectively, indicating that the massive infiltration of mononuclear cells upon liver injury was significantly diminished upon hEnSC transplantation (Fig. 2a). Flow cytometry analyses were performed using anti-CD68 and anti-CD163 antibodies to identify subpopulations of monocytes/MoMFs/Kupffer cells. The percentage of C68^+^CD163^+^ cells, which represent the immunoregulatory (M2) subpopulations of MoMFs/Kupffer cells,^21,22^ in the hEnSC-transplanted group was higher than that in the sham ALF group (Fig. 2b; Supplementary information, Fig. S6a). In addition, qRT-PCR analyses performed on monocytes/MoMFs/Kupffer cells isolated from hEnSC-transplanted livers revealed a dramatic downregulation of the acute inflammatory factor *Tnfα* and inducible nitric oxide synthase (*iNos*) as well as a marked upregulation of the key anti-inflammatory cytokine *Il10*^23–26^ and the hepatoprotective factor *Il6*^27–29^ which is known to counter-regulate TNF*α* signaling^30^ (Fig. 2c). These results suggest that the activation state of MoMFs/Kupffer cells and the general hepatic immune micromilieu might be skewed towards an anti-inflammatory state after hEnSC transplantation.

We next investigated the adaptive immune cell populations in the liver upon hEnSC transplantation. Similar to macrophages, hepatic T cells display remarkable heterogeneity that is essential for their proinflammatory and anti-inflammatory functions in response to liver damage. The lymphocyte cell counts in both the sham and the hEnSC-transplanted ALF groups were markedly elevated compared to those of the healthy group, with the ones of the hEnSC-transplanted group being significantly lower than those of the sham group (Fig. 2d). Helper T (Th) cells and regulatory T (Treg) cells, which are both derived from common naïve T cells, are often tightly associated and play distinct roles in inflammation and its resolution during tissue injury.^31,32^ Therefore, we focused our investigation on proinflammatory interferon-γ (IFNγ) or IL17-producing helper T (Th1 and Th17, respectively) and immunosuppressive Treg cells in ALF livers treated with hEnSCs or PBS. As expected, flow cytometry analyses revealed that the proportions of CD4^+^IFNγ^+^ Th1, CD4^+^IL17A^+^ Th17 (Fig. 2e; Supplementary information, Fig. S6b), and CD4^+^CD25^+^FOXP3^+^ Treg cells (Fig. 2f; Supplementary information, Fig. S6c) increased in the PBS-treated ALF rat livers but returned to the levels seen in healthy livers in the hEnSC-transplanted group. The percentage regression of both the proinflammatory T helper and the anti-inflammatory Treg cells upon hEnSC transplantation may represent the tight balance of these two cohorts of CD4^+^ T cells.^33,34^ These data indicate that hEnSC transplantation also strongly affected the adaptive immune system and thereby promoted quick resolution of inflammation in the liver.

Altogether, these lines of evidence support a strong immunomodulatory phenotype of hEnSCs in the injured liver. It is likely that the phenotypic and functional changes observed in the innate immune cells, in particular the liver MoMFs and Kupffer cells, induced the subsequent changes of the adaptive immune cells.^5^

### ScRNA-seq analyses reveal population heterogeneity and phenotypic changes upon hEnSC transplantation in liver MoMFs and Kupffer cells

To overcome the difficulties of analyzing rat cells caused by the lack of well-defined markers and to decipher the mechanism by which hEnSC transplantation modulates the central immune compartment of monocytes, macrophages and Kupffer cells in the context of ALF, scRNA-seq was performed to delineate population heterogeneity and global transcriptomic changes upon hEnSC transplantation. Briefly, the mixed cell populations mainly containing monocytes, MoMFs and Kupffer cells were isolated from three replicate rats of each group (healthy, PBS-treated ALF and hEnSC-transplanted ALF) and subjected to 10× genomic sequencing. A total of 12,177 cells were analyzed, with the outliners (detected genes < 500) removed. Further selection for monocyte/macrophage/Kupffer cells was carried out according to the expression of *Cd68*, and 10,927 cells (healthy, 3041 cells; PBS-treated ALF, 4274 cells; hEnSC-transplanted ALF, 3612 cells) were retained.

**Fig. 3:**
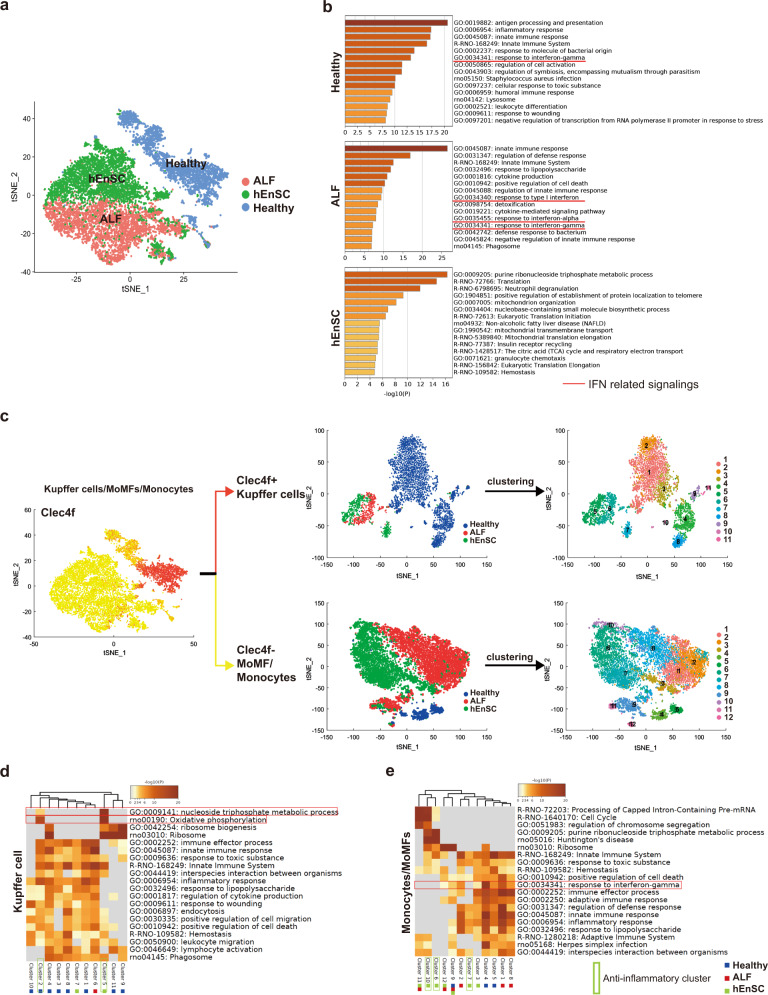
Single-cell transcriptomic analyses of liver macrophages reveal phenotypical and functional changes in cell subpopulations. **a** t-SNE plot of the monocyte/MoMF/Kupffer cell populations isolated on day 3 post drug administration and transplantation from healthy (blue), PBS-treated ALF (red) and hEnSC-transplanted ALF (hEnSC) (green) rat livers. For each sample of scRNA-seq, the monocyte/MoMF/Kupffer cell populations isolated from 3 independent animals under identical treatment were combined. Cells other than monocytes/MoMFs/Kupffer cells were removed by pan-macrophage marker *Cd68* before analysis. Batch effect was corrected based on the expression of the house-keeping genes. **b** Top 15 GO terms enriched in each indicated condition. IFN signaling-related GO terms are underscored with red lines. **c** t-SNE plots showing the separation and clustering of Kupffer cell (*Clec4f*^*+*^) and MoMF/monocyte (*Clec4f*^–^) populations based on the expression of Kupffer cell marker *Clec4f*. **d**, **e** GO analysis of cell subpopulations in Kupffer cell and infiltrated monocyte/MoMF cohorts as shown in **c**. The sample constitutions of each cluster are marked by color-coded squares. Blue: healthy group; Red: PBS-treated ALF group; Green: hEnSC-transplanted ALF group. Anti-inflammatory clusters are marked with green frames. See also Supplementary information, Figs. S8–S11.

To obtain an in-depth understanding of the transcriptomic landscapes of monocytes/MoMFs/Kupffer cells from the three groups, all cells were projected into a single 2D t-stochastic neighbor embedding (t-SNE) plot.^35^ Strikingly, the cells of each group were distinctly clustered when batch effects were removed depending on the expression of housekeeping genes.^36^ The cells from the healthy group completely separated from the cells of the ALF or hEnSC groups, while the cells from the latter two groups juxtaposed with subtle overlaps (Fig. 3a). Unsupervised clustering analysis was performed with SEURAT^37^ to further dissect the differential gene expression patterns across the cell populations, which led to a heatmap of the top 15 differentially expressed genes (DEGs) in the cells ordered in three groups (Supplementary information, Fig. S8a). Three gene clusters were identified, and gene ontology (GO) analysis revealed that cluster 2 harbored a panel of interferon-stimulated genes (ISGs), including *Mx1*, *Irf7*, *Cxcl10*, *Isg15*, *Mx2*, *Eif2ak2*, *Sp100*, *Ifit2*, *Ifit3* and *Ifitm3*,^38,39^ which were upregulated in the cells from the PBS-treated ALF (ALF) group as compared with those in the cells from both the healthy and the hEnSC-transplanted (hEnSC) groups (Fig. 3b; Supplementary information, S8b), indicating that the activation of IFN signaling in monocytes/MoMFs/Kupffer cells from ALF livers was effectively suppressed upon hEnSC transplantation.

To further demarcate the population heterogeneity of the monocytes, macrophages and Kupffer cells across the three groups and thereby to dissect the pro-/anti-inflammatory roles of cell subpopulations in the context of ALF, we first used *Clec4f* as a marker to distinguish Kupffer cells (*Clec4f*^*+*^) from the infiltrated monocytes and MoMFs (*Clec4f*^–^)^40,41^ and then performed unsupervised clustering analysis with SEURAT to delineate the subpopulations of each cell cohort (Fig. 3c). The expression pattern of *Clec4f* across the cells overlapped with those of other Kupffer cell markers, including *Marco* and *Vsig4* (*CRIg*) (Supplementary information, Fig. S9a), which further confirmed the classification. The sizes of the *Clec4f*^*+*^ Kupffer cell populations in both the ALF (406 cells) and hEnSC (474 cells) groups were much smaller than that of the healthy group (2542 cells), while the sizes of the *Clec4f*^–^ monocyte/MoMF populations were much larger in both the ALF (3868 cells) and the hEnSC (3138 cells) groups than that of the healthy group (499 cells) (Fig. 3c), which is consistent with the fact that during injury, the monocytes recruited from the bone marrow often greatly exceed the population of resident macrophages.^7,42^

In the *Clec4f*^+^ Kupffer cell cohort, 11 subpopulations were identified across the three scenarios (Fig. 3c). Cluster 6 mainly contained the cells of the ALF group, clusters 5 and 7 represented the cells from the hEnSC group, and the rest of the clusters harbored the cells from the healthy group. GO analysis of the DEGs revealed that cluster 2 of the healthy group might represent the immunoregulatory subset, as evidenced by high expression of *Marco*, *Cd163* and *Mrc1* (Supplementary information, Fig. S9b), which are markers for M2 Kupffer cells.^7^ Similarly, cluster 5 of the hEnSC group might also represent the immunoregulatory subset based on the expression of *Marco*, *Cd163*, and *Ccl24*^43^ (Supplementary information, Fig. S9b). In addition, both cluster 2 (healthy group) and cluster 5 (hEnSC group) were enriched for the GO terms “oxidative phosphorylation” and “nucleoside triphosphate metabolic process” indicative of the M2-like metabolic signature,^44^ and these two clusters were negative for “lymphocyte activation” (Fig. 3d). In particular, cluster 5 (hEnSC group) was negative for the GO terms “innate immune response”, “inflammatory response”, “immune effector response”, “response to lipopolysaccharide”, “regulation of cytokine production” or “response to wounding”, suggesting that these cells have an immunoregulatory (M2-like) nature. Notably, the cells of cluster 6 (ALF group) were enriched for ISGs, including *Mx1* and *Irf7*; in contrast, these genes were significantly downregulated in clusters 5 and 7 from the hEnSC group (Supplementary information, Fig. S10), suggesting inhibition of IFN signaling in the Kupffer populations upon hEnSC transplantation.

In the *Clec4f*^–^ monocyte/MoMF cohort, 12 cell subsets were identified (Fig. 3c). Importantly, the expression of a spectrum of ISGs, including *Ifit2*, *Ifit3*, *Irf7*, *Isg15* and *Mx1*, was significantly downregulated in the cell clusters 3, 6, 7 and 10 from the hEnSC group compared to the cell clusters 1, 2 and 8 from the ALF group (Supplementary information, Fig. S11), indicating that the IFN signaling activated in the monocytes/MoMFs from ALF livers was suppressed after hEnSC treatment. GO analysis further confirmed that the term “response to interferon-gamma” was negatively regulated in clusters 6, 7 and 10 from the hEnSC group (Fig. 3e), and these cell subsets were also negative for a panel of proinflammatory terms, including “adaptive immune response”, “inflammatory response”, “immune effector response”, “response to lipopolysaccharide” and “regulation of defense response”. These data suggest that the monocyte/MoMF subpopulations were also reprogrammed to immunoregulatory states after hEnSC transplantation.

Collectively, these data clearly demonstrated at single-cell resolution that the monocyte/MoMF/Kupffer cell compartment underwent dramatic phenotypic and functional changes from a proinflammatory to immunoregulatory/reparative states upon hEnSC transplantation in ALF rats and that the downstream target genes of IFN signaling were significantly downregulated in these cell populations.

### hEnSCs modulate macrophages by inhibiting IFN signaling

**Fig. 4:**
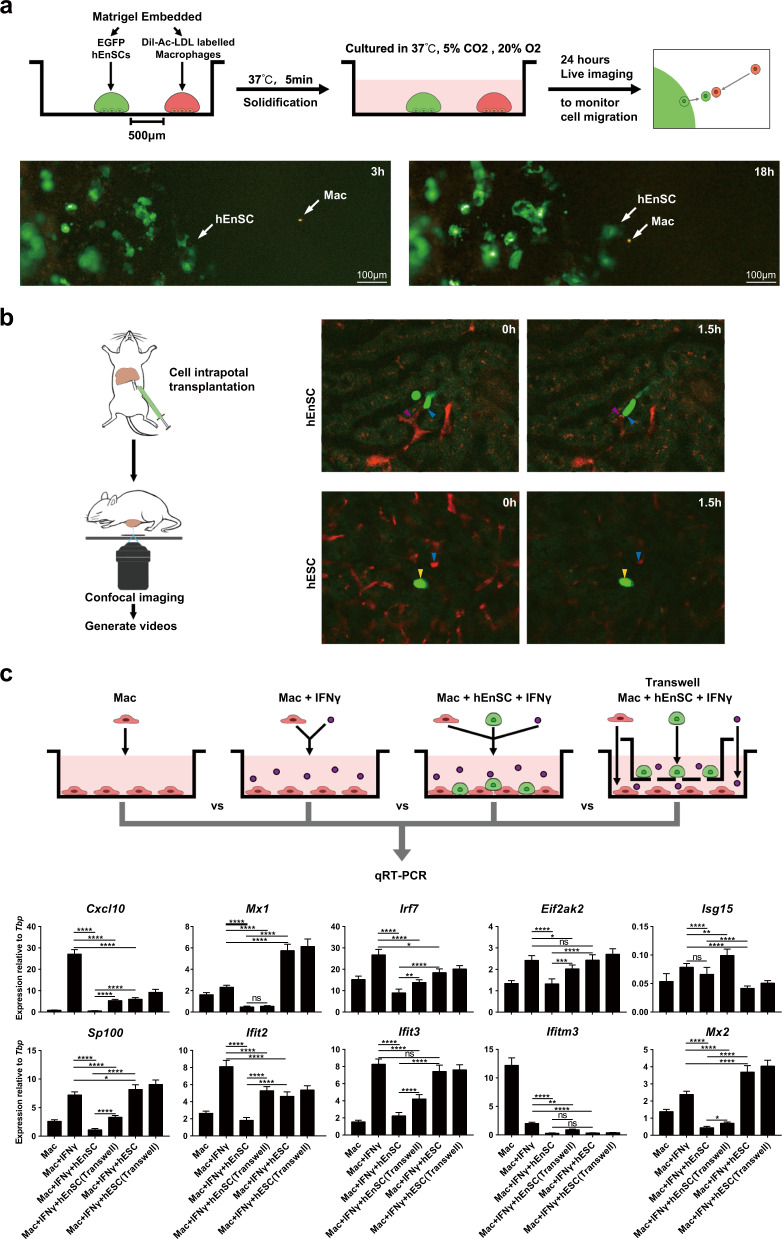
hEnSCs modulate macrophages by inhibiting IFNγ signaling. **a** Upper panel: schematic diagram of in vitro migration assay; lower panel: representative fluorescence images captured by live imaging system showing the movement of EGFP-hEnSCs and DiI-Ac-LDL-labeled MoMFs/Kupffer cells. **b** Left: schematic diagram of intravital microscopy used to monitor in vivo interactions between hEnSCs and MoMFs/Kupffer cells; right: representative fluorescence images captured by live confocal microscope showing the interactions between EGFP-hEnSCs (pink arrows) and F4/80-PE-stained MoMF/Kupffer cells (blue arrows), and the interactions between EGFP-hESCs (yellow arrows) and F4/80-PE-stained Kupffer cells (blue arrows). **c** qRT-PCR showing expression of the indicated ISGs in the monocyte/MoMF/Kupffer cell populations cocultured with or without hEnSCs. The monocyte/MoMF/Kupffer cell populations were isolated from ALF rat livers at 24 h post D-GalN treatment, and were cocultured for 3 days with an initial seeding ratio of 1:1 in the presence or absence of rat recombinant IFNγ. Values were determined relative to *Tbp*. *n* = 3 independent samples for each group. Tukey’s multiple comparisons test was used to calculate significance between groups. ns, no significance; *P < 0.05; **P < 0.01; ***P < 0.001; ****P < 0.0001. See also Supplementary information, Figs. S12, S13, Videos S2–S4.

Given the dramatic changes in the gene expression profiles of macrophages/Kupffer cells after hEnSC transplantation, we hypothesized that hEnSCs might directly modulate the activation state of liver macrophages. First, to test whether EGFP-hEnSCs and the DiI-Ac-LDL-labeled MoMFs/Kupffer cells isolated from rat livers were able to interact directly, these two types of cells were seeded apart from each other in the same well of a dish and monitored for migration with a live imaging system (Fig. 4a). Migration and direct cell–cell contacts between hEnSCs and macrophages were observed (Fig. 4a; Supplementary information, Video S2). Moreover, using intravital microscopy we monitored the in situ interactions between EGFP-hEnSCs (or EGFP-hESCs) and the fluorescently labeled Kupffer cells/MoMFs right after transplantation for 1–3 h. Strikingly, intimate and dynamic interplays were clearly observed between hEnSCs (but not hESCs) and Kupffer cells/MoMFs that nested in the liver sinusoidal capillaries (Fig. 4b; Supplementary information, Videos S3, S4). Interestingly, some of Kupffer cells/MoMFs appeared to be engulfing hEnSCs (Supplementary information, Video S3), suggesting that hEnSCs which are negative for CD47 (Supplementary information, Fig. S1b, c) might be cleared through the efferocytosis of these cells, a fact that may in turn impose effects on the phenotype of Kupffer cells/MoMFs. These observations indicate that hEnSCs and macrophages/Kupffer cells are capable of attracting each other both in vitro and in vivo, possibly through chemokine signaling.

It has been reported that IFNγ signaling plays essential roles in monocyte recruitment, maintenance of inflammation and aggravation of liver damage.^45,46^ Since the scRNA-seq data revealed marked downregulation of IFN signaling (Supplementary information, Figs. 3b, S8, S10, S11) in both Kupffer cells and the infiltrated monocyte/MoMF populations from the hEnSC-transplanted ALF livers, we next tested whether hEnSCs were able to suppress the activation of this signaling in vitro. Indeed, using rat-specific primers, qRT-PCR analyses on the ex vivo cultures of Kupffer cells/MoMFs/monocytes isolated from healthy livers revealed significant downregulation of a panel of ISGs in the presence of hEnSCs and exogenous IFNγ (Fig. 4c). Notably, the downregulation of a subset of ISGs in the presence of hEnSCs was less prominent when Transwells were used (Fig. 4c), which suggested that either direct cell–cell contact or juxtaposition might be required for efficient suppression.

It is conceivable that the transplanted hEnSCs may modulate monocyte/MoMF/Kupffer cells either by secreting paracrine factors and/or by direct cell–cell contact. To elucidate the secretome of hEnSCs and compare it with that of hMSCs, a cytokine array was utilized to assay the supernatants collected from the cell cultures. Notably, hEnSCs secreted the proinflammatory cytokines GM-CSF, CXCL8, IL1β, IL11 and IL6^5,7,47^ at levels significantly lower than those observed in hMSCs (Supplementary information, Fig. S12), suggesting a more efficient immunomodulatory function of hEnSCs. On the other hand, hEnSCs secreted Th2 cytokines, including IL4 and IL13, at levels comparable to those of hMSCs (Supplementary information, Fig. S12). In addition, qRT-PCR performed on samples from ALF rat livers transplanted with either hEnSCs or hMSCs allowed us to obtain a panoramic view of the expression of inflammation-related genes in whole livers. Compared to ALF livers, hEnSC-transplanted livers showed significant upregulation of *Il10*, a critical anti-inflammatory mediator^7,23,25,26,48^ and the anti-inflammatory factor *Il2*, as well as downregulation of the proinflammatory factor *Il1β* (Supplementary information, Fig. S13). In addition, hEnSC-transplanted livers expressed significantly more of the anti-inflammatory factor *Il10* and less of the pro-inflammatory factors *Il1β*, *Gm-csf*, *Il12p70* than hMSC-transplanted livers^49^ (Supplementary information, Fig. S13). The upregulation of *Il10* and other anti-inflammatory cytokines in the liver is likely a result of the downregulation of IFNγ signaling in the Kupffer cell/monocyte/MoMF and other IL10-expressing cell populations, as it has been established that IFNγ can directly suppress the IL10-STAT3 axis by inhibiting both Toll-like receptor (TLR)-induced gene expression and downstream STAT3 signaling.^44^

In addition, we also observed that the anti-inflammatory cytokine *Il10* was upregulated while the pro-inflammatory cytokines *Tnfa* and *Il6* were significantly downregulated in the MoMFs/Kupffer cells isolated from hEnSC-transplanted APAP-induced ALF animals (Supplementary information, Fig. S4c), which suggests a mechanism similar to that underlies D-GalN model.

These data suggest that the ability of hEnSCs to suppress IFNγ signaling might account for the elevated expression of immunoregulatory cytokines including IL10 as well as the downregulation of proinflammatory cytokines, which revealed differential immunomodulatory effects of hEnSCs versus hMSCs in the context of ALF.

### hEnSC-derived cystatin SN suppresses IFNγ signaling in macrophages and kupffer cells

**Fig. 5:**
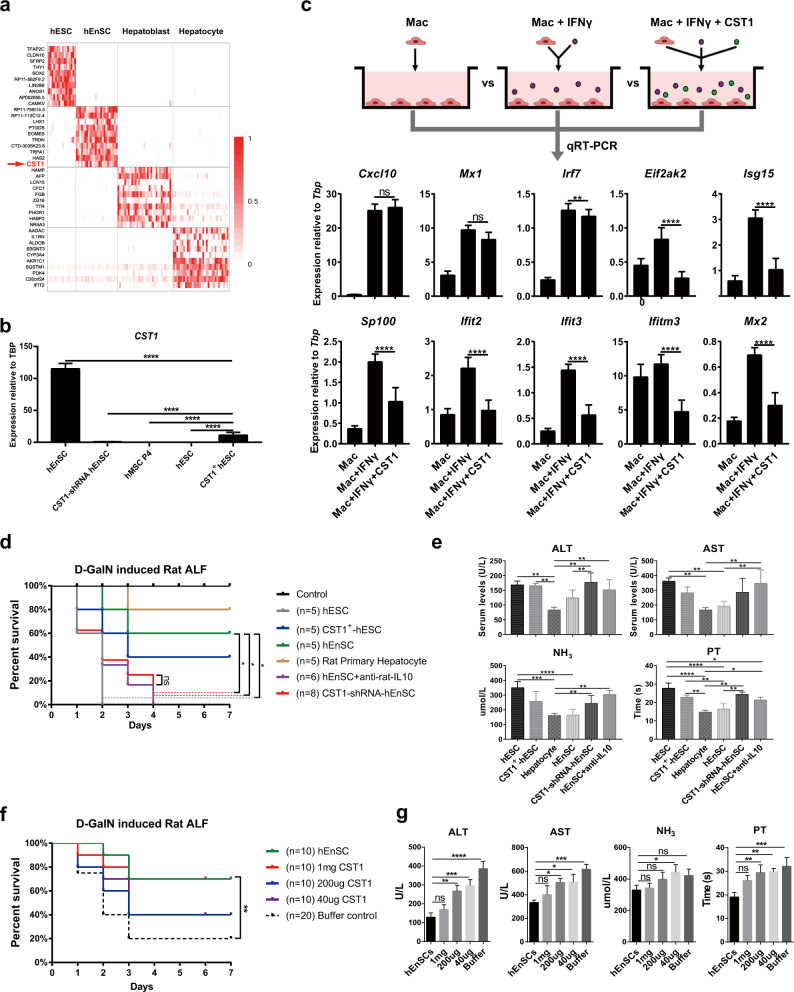
hEnSC-secreted CST1 is responsible for the inhibition of IFNγ signaling. **a** Heatmap of the top 10 DEGs identified by scRNA-seq (SMART-seq2) across the cell populations of hEnSCs, hESCs, hESC-derived hepatoblasts or hepatocytes, showing the exclusive expression of CST1 in hEnSCs. **b** qRT-PCR showing expression of CST1 in the cell lines of H9 hESCs (hESC), CST1-overexpressing H9 hESCs (CST1^+ ^hESC), human umbilical mesenchymal stem cells (hMSC P4), H9 hESC-derived EGFP-hEnSCs (hEnSC), and hEnSCs with shRNA-mediated knockdown of CST1 (CST1-shRNA hEnSC). Values were determined relative to *TBP*; *n* = 3 independent samples for each group. **c** qRT-PCR showing expression of indicated ISGs in monocyte/MoMF/Kupffer cell populations that were isolated from ALF rat livers 24 h post D-GalN administration and cultured in vitro for 3 days in the presence or absence of CST1 protein (500 ng/mL) and/or recombinant rat IFNγ (100 ng/mL). Values were determined relative to *Tbp*; *n* = 3 independent samples for each group. **d** Kaplan–Meier survival curves of the ALF rats transplanted with H9 hESCs (hESC), CST1-overexpressing hESCs (CST1^+^ hESC), rat primary hepatocytes, hEnSCs, hEnSCs with shRNA-mediated knockdown of CST1 (CST1-shRNA hEnSC) and hEnSCs + anti-rat IL10 antibody (1 mg/rat) (hEnSC + anti-rat-IL10). **e** Serum levels of ALT, AST, NH_3_ and PT detected in the indicated groups as shown in **d**. *n* = 3 for each group. Blood samples were collected 3 days post D-GalN administration. **f** Kaplan–Meier survival curves of the ALF rats transplanted with different doses of CST1 protein. 1 mg/200 μg/40 μg CST1: 1 mg or 200 μg or 40 μg CST1 protein per rat; Buffer control: PBS + 10% glycol. **g** Serum levels of ALT, AST, NH_3_ and PT detected in the indicated groups as shown in **f**. *n* = 3 for each group. Blood samples were collected 3 days post D-GalN administration. *P < 0.05; **P < 0.01; ***P < 0.001; ****P < 0.0001. Tukey’s multiple comparisons test was used in **b**, **c**, **e** and **g**, and Log-rank (Mantel-Cox) test was used in **d** and **f**. See also Supplementary information, Fig. S14.

To identify the hEnSC-derived regulators that inhibit IFNγ signaling in monocytes/MoMFs/Kupffer cells, we mined the data from scRNA-seq analyses performed on hEnSCs, hESCs and hESC-derived hepatoblasts or hepatocytes. Among the panel of DEGs, CST1, a cysteine proteinase inhibitor that belongs to type 2 cystatin family and is implicated in immune defense against bacterial, parasitic and viral infections,^50–52^ attracted our attention and was extrapolated to be the potential IFN regulator as it was preferentially expressed in hEnSCs rather than other cell types including hMSCs (Fig. 5a, b). In line with this speculation, we did observe significant downregulation of ISGs when we applied human recombinant CST1 proteins instead of hEnSCs to the ex vivo culture of monocyte/MoMF/Kupffer cells isolated from ALF rat livers (Fig. 5c).

Then we set out to determine whether CST1 played an essential role in the alleviation of hepatic injuries in ALF animals. By knocking down CST1 expression with lentiviral CST1-shRNA in hEnSCs (CST1-shRNA-hEnSCs) (Fig. 5b) and intraportally transplanting the cells into D-GalN-induced ALF rats, we found that all the animals receiving CST1-shRNA-hEnSCs died within 4 days (Fig. 5d). The blood samples from the CST1-shRNA-hEnSC-transplanted group showed significantly higher levels of ALT, NH_3_ and PT than those from the hEnSC- or rat primary hepatocyte-transplanted groups (Fig. 5e). These results clearly demonstrated that the knockdown of CST1 in hEnSCs abolished the curative effects of these cells on ALF.

Next, we investigated whether CST1 overexpression in CST1-negative cells can alleviate liver injury. Human ESCs were chosen for overexpression, as they do not express CST1 and show no ameliorative effect on ALF (Figs. 1e, 5d). Notably, 40% of rats transplanted with CST1-overexpressing hESCs (CST1^+^ hESCs) survived at 7 days post drug administration, a survival rate better than that of the unmodified hESCs yet inferior to those of hEnSCs (60%) or rat primary hepatocytes (80%) (Fig. 5d). This result is consistent with the patterns of liver injury parameters across the groups (Fig. 5e). The significantly lower expression level of CST1 in CST1^+^ hESCs (Fig. 5b) than in hEnSCs and/or the lack of intimate interaction between hESCs and MoMFs/Kupffer cells (Fig. 4b; Supplementary information, Videos S3, S4) may account for the suboptimal curative effect of CST1^+^ hESCs on ALF.

Since IFNγ has inhibitory effects on IL10 production and STAT3 downstream signaling,^44^ we hypothesized that IL10 might act as the key direct downstream effector of CST1-mediated inhibition of IFN signaling and the consequent alleviation of hepatic injury, which was supported by the upregulation of IL10 in both the monocyte/MoMF/Kupffer cell population and the whole liver upon hEnSC transplantation (Fig. 2c; Supplementary information, Fig. S13). Notably, we observed that all of the rats receiving hEnSCs + anti-IL10 antibodies died within 4 days (Fig. 5d) and these animals showed significantly higher levels of ALT, AST, NH_3_ and PT as compared with the groups receiving rat primary hepatocytes or hEnSCs (Fig. 5e). In addition, no difference in hepatic injury indexes was observed between the hEnSC + anti-IL10 and CST1-shRNA-hEnSC groups (Fig. 5e). These data demonstrated that the inhibition of IL10 signaling completely abolished the efficacy of hEnSCs in treating ALF.

Finally, to evaluate the potential of CST1 per se as a therapeutic candidate for ALF, we treated D-GalN-induced ALF rats with recombinant CST1 protein at three dosages (1 mg, 200 μg or 40 μg per rat) by portal vein injection. The survival rate (70%) of the rats receiving the highest dosage approximates that of the rats transplanted with hEnSCs, while the rates were reduced to 40% in the ALF groups receiving lower dosages (Fig. 5f). The patterns of the liver injury parameters were consistent with the survival rates (Fig. 5g). These data indicate that CST1 alone has the potential to alleviate inflammatory hepatic injuries.

### CST1 suppresses IFNγ by directly binding to IFNGRs

**Fig. 6:**
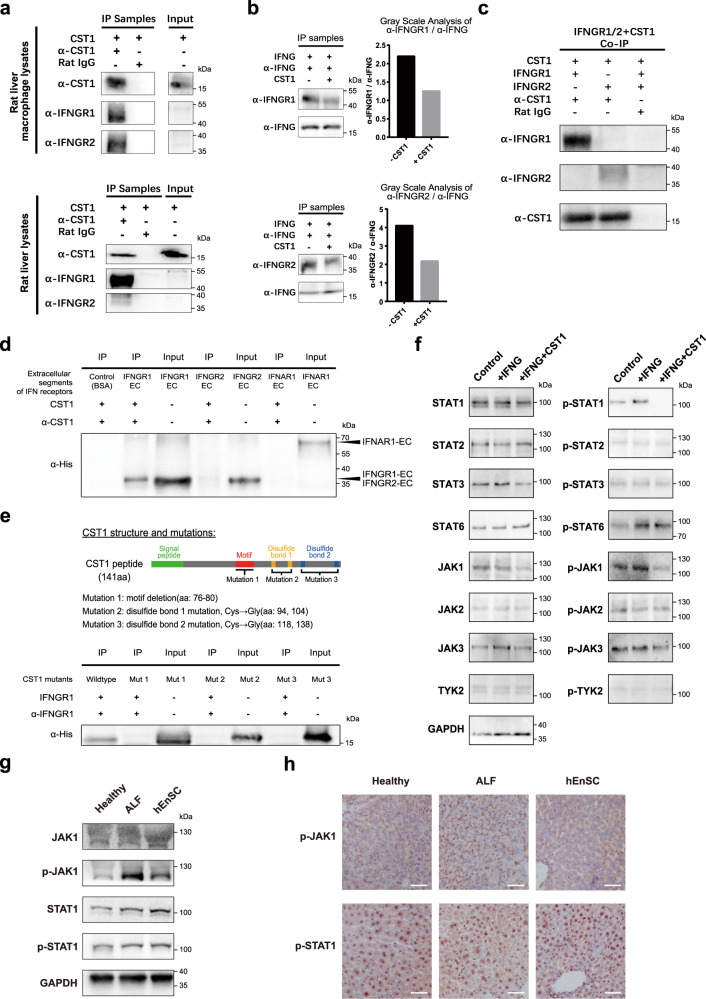
CST1 inhibits IFNγ signaling via interaction with IFNγ receptors. **a** Interactions between CST1 and IFNGR1 or IFNGR2 in the cell lysates of the monocyte/MoMF/Kupffer cell populations isolated from ALF rats (upper panel) and in the ALF rat liver tissue lysates (lower panel). Co-immunoprecipitation was performed using recombinant human CST1 protein and anti-CST1 antibody (or Rat IgG as negative control) with cell or tissue lysates, followed by western blotting using anti-CST1, anti-IFNGR1 or anti-IFNGR2 antibodies. **b** Competition assay showing the interference of ligand–receptor binding of IFNG–IFNGR1/2 by CST1 proteins. Immunoprecipitation was performed using recombinant IFNG, rat monocyte/MoMF/Kupffer cell lysates (as a source for IFN receptors) isolated from ALF rats, in the presence or absence of recombinant CST1, followed by western blotting using anti-IFNGR1 (upper panels) or anti-IFNGR2 antibodies (lower panels), with gray-scale analyses showing the changes in ratios of either α-IFNGR1/α-IFNG or α-IFNGR2/α-IFNG upon the administration of CST1 proteins. **c** Direct interactions between CST1 and IFNGR1 or IFNGR2. Immunoprecipitation was performed using recombinant proteins of CST1, IFNGR1, IFNGR2 and anti-CST1 antibody (or Rat IgG as negative control), followed by western blotting using anti-CST1, anti-IFNGR1 or anti-IFNGR2 antibodies. **d** Direct interactions between CST1 and extracellular segments of IFNGR1 or 2. The His-tagged extracellular segments (EC) of rat IFNGR1 or 2 were expressed by *E. coli* and purified for the immunoprecipitation that used anti-CST1 antibody, in the presence or absence of recombinant CST1. Anti-His antibody was used for western blotting. IFNGR1-EC, IFNGR1 extracellular segment (aa: 1–241); IFNGR2-EC, IFNGR2 extracellular segment (aa: 1–240); IFNAR1-EC, IFNAR1 extracellular segment (aa: 1–431). **e** CST1 domains essential for IFNGR1 binding. Upper panel: three mutants of human CST1 were generated by the deletion of the only motif (Mut 1), or by the disruption of two disulfide bonds (Mut 2 or Mut 3) respectively, and were His-tagged and expressed in *E. coli*. Lower panel: immunoprecipitation performed using wild-type or CST1 mutant proteins. Anti-His antibody was used for western blotting. **f** Changes in the phosphorylation status of the IFN-related effectors of JAK/STAT family in the presence of CST1. The cell lysates of the monocyte/MoMF/Kupffer cell populations isolated from ALF mice were incubated with IFNγ (IFN) or IFN + CST1 or without IFN or CST1 (control), and immunoblotted using the antibodies that detected either total proteins or the phosphorylated forms of STAT1, 2, 3 and 6, JAK1, 2 and 3 and TYK2. **g** Western blotting on the total proteins and the phosphorylated forms of JAK1 and STAT1 using liver tissue lysates prepared from healthy, ALF and hEnSC-treated ALF (hEnSC) mice (day 2 post D-GalN treatment). **h** Immunohistochemistry of phosphorylated JAK1 and STAT1 in the mouse liver tissue sections prepared from healthy, ALF and hEnSC-treated ALF (hEnSC) mice (day 2 post D-GalN treatment). Scale bars, 50 μm.

To dissect the mechanism by which CST1 inhibited IFNγ signaling, we focused on CST1 interaction with IFN signaling-related proteins. Intriguingly, the co-immunoprecipitation (Co-IP) assay using rat liver macrophage lysate revealed physical interactions between CST1 and IFNGR1 or IFNGR2 (Fig. 6a), which is a novel finding that suggests a role of CST1 in interrupting the binding of IFNγ to its receptors. This was further supported by the competition Co-IP assay performed on the macrophage cell lysate in the presence of recombinant CST1 protein, which demonstrated significant reductions in the ratios of IFNGR1/IFNγ or IFNGR2/IFNγ (Fig. 6b). In addition, CST1 and IFNGR1/2 proteins were used to replace the cell lysates to further confirm the direct interactions among them (Fig. 6c). To determine the specific domains of IFNGRs to which CST1 is able to bind, the His-tagged extrapolated extracellular parts of IFN receptors (IFNGR1-EC, IFNGR2-EC and IFNAR1-EC) were prepared with the protein expression system and assayed by co-IP which revealed that CST1 was able to directly bind IFNGR1-EC or IFNGR2-EC, but not IFNAR1-EC (Fig. 6d). Lastly, to determine the domains of CST1 essential for its binding with IFNGRs, either the only motif (aa: 76–80) besides the signal peptide was deleted (Mut 1) or the two disulfide bonds (aa: 94,104 and 118,138) were mutated from cystein to glycine,  respectively (Mut 2 and Mut 3), and the three mutants were tested by co-IP with IFNGR1, respectively. The deletion of the motif (aa: 76–80) and the mutations of the disulfide bonds completely disabled the binding ability of CST1 to IFNGR1/2 (Fig. 6e).

Next, the phosphorylation statuses of various JAK and STAT proteins that are known to be important in signal transduction^53^ were interrogated in the cultured Kupffer cell/MoMFs or in the liver tissue lysates in the presence or absence of exogenous CST1 proteins. Notably, the protein levels of the phosphorylated JAK1 (p-JAK1) and STAT1 (p-STAT1), the well-known downstream effectors of IFN receptors, were elevated upon IFNγ stimulation as expected, and were significantly downregulated when CST1 were added in the cultured Kupffer cell/MoMFs (Fig. 6f). However, only p-JAK1, but not p-STAT1, was downregulated in the liver tissue lysates (Fig. 6g) or sections (Fig. 6h) 24 h post hEnSC treatment, which might represent the differential reactions of various liver cell populations to hEnSC (or CST1) treatment. These results confirmed that CST1 selectively inhibited IFNGR1 and IFNGR2 downstream signaling events.

In conclusion, CST1 inhibits IFNγ signaling probably by directly binding to the extracellular domains of IFNGRs and competing with IFNγ, which results in the downregulation of p-JAK1, p-STAT1 and the downstream ISGs. The disulfide bonds of CST1 are indispensable for the binding.

## Discussion

ALF is a life-threatening disease with very limited treatments. As the most effective therapy to date, orthotopic liver is suitable for less than 10% of all ALF patients, and its application is severely restricted due to donor shortages, life-long requirements for immune suppression and surgical challenges.^1,2^ Given the immune dysregulatory nature of ALF,^54^ cell therapies incorporating stem cells that possess immunomodulatory functions appear to be promising alternative treatments for a broad range of patients.^55^ Here, we report a novel and effective stem cell therapy for ALF using nontumorigenic hEnSCs for transplantation.

Notably, hEnSCs transplanted into the liver are able to effectively reverse hepatic injury in multiple rodent and swine ALF models. It has been reported that MSC transplantation also effectively ameliorates liver injury, by releasing trophic and immunomodulatory factors and by inducing apoptosis or anergy of T cells.^12,27,56–58^ Similar to MSCs, hEnSCs exhibit low immunogenicity for T cells as they do not express HLAs, which allows for safe allogeneic transplantation without acute inflammation, confirmed by the observation that CRP levels rapidly returned to normal within a week post drug administration (Fig. 1h). Nevertheless, hEnSCs appear to regulate the immune microenvironment in a manner distinct from MSCs, which is demonstrated by the unique ability of hEnSCs to suppress IFNγ signaling in MoMF/Kupffer cell populations through the secretion of CST1 which is not expressed by hMSCs (Fig. 5b). In addition, hEnSCs have some salient advantages over MSCs. First, hEnSCs are nontumorigenic in immunocompromised animals, as the normal in vivo micromilieus of the immunocompromised animals favors rapid differentiation rather than proliferation of these cells^15–17^ (Supplementary information, Fig. S1e); while in the ALF context of wild-type animals, these cells appear to be rapidly cleared within a week (Fig. 1d), probably by macrophages and/or NK cells as they do not express CD47 or HLAs (Supplementary information, Fig. S1a–c). Second, unlike hMSCs, which are prone to unwanted myofibroblast differentiation that might aggravate fibrosis, hEnSCs are endoderm restricted. These advantages make hEnSCs more appropriate as a potential cell-based drug for treating ALF than MSCs.

As it has been widely accepted that the fine-tuned inflammatory response and its timely resolution are the central events that orchestrate tissue repair after injury,^5^ in this study we focus on the hepatic immune microenvironment instead of the damage and regeneration of hepatocytes. We investigated the innate and adaptive immune responses after hEnSC transplantation, as the ultimate outcome of the intrahepatic immune response is determined by the functional diversity of liver macrophages and by the delicate balance between proinflammatory and anti-inflammatory T cell populations.^7,31,32^ First, we found that the monocytes/MoMFs recruited from the circulation dominated the liver macrophage population (Fig. 2a) and that the number of liver macrophages expanded by 4-fold in the ALF livers relative to the healthy livers but decreased by 2-fold upon hEnSC transplantation, suggesting that hEnSC treatment efficiently suppressed the recruitment of monocytes and the proliferation of Kupffer cells. Second, the phenotypic and functional reprogramming of liver macrophages after hEnSC transplantation was clearly demonstrated by the higher percentage of CD163^**+**^ immunosuppressive (M_reg_) subpopulations of MoMFs/Kupffer cells in the hEnSC-transplanted group than in the sham group (Fig. 2b) and by the dramatic downregulation of the acute inflammatory factors *Tnfα* and *iNos* as well as the marked upregulation of the key anti-inflammatory cytokine *Il10* in liver macrophages isolated after hEnSC treatment (Fig. 2c). Third, the scRNA-seq analyses exquisitely dissected the population heterogeneity in *Clec4f*^+^ Kupffer cells and *Clec4f*^–^ monocytes/MoMFs isolated from the liver and unequivocally revealed the changes in the liver macrophage activation state after hEnSC transplantation. Along with the typical proinflammatory (M1) subsets, the immunoregulatory (M2 or M_reg_) cell subsets in both healthy and hEnSC-transplanted ALF livers were identified by the expression of anti-inflammatory factors and the M2 metabolic signature^44^ (Fig. 3c–e; Supplementary information, Figs. S9b, S10, S11). Intriguingly, the subpopulations with mixed M1 and M2 markers were also detected in all three groups (Fig. 3d, e; Supplementary information, Figs. S10, S11), representing the spectrum of macrophage activation states that extend beyond the M1/M2 dichotomy.^59,60^ Fourth, scRNA-seq analyses also revealed the marked suppression of IFN signaling after hEnSC transplantation in both *Clec4f*^+^ Kupffer cells and *Clec4f*^–^ monocytes/MoMFs (Fig. 3b; Supplementary information, Figs. S8, S10, S11). Although we cannot rule out the possibility that the monocytes may adopt a Kupffer cell phenotype (including upregulation of *Clec4f* and other Kupffer cell markers) upon the loss of Kupffer cells, this limitation will not impact on our conclusion as the general activation states of MoMFs/Kupffer cells as a whole were biased towards anti-inflammatory ones upon hEnSC transplantation (Figs. 2b, 3; Supplementary information, Fig. S6a). Fifth, we found that the proportions of both proinflammatory Th1 and Th17 cells and anti-inflammatory Treg cells increased in the PBS-treated ALF livers and returned to the levels of healthy livers upon hEnSC transplantation (Fig. 2e, f), which may represent the tight balance of these two cohorts of CD4^+^ T cells^33,34^ and suggests that the influence of hEnSCs on T cell populations may be secondary to their direct effect on MoMFs/Kupffer cells. Finally, we also analyzed the neutrophil compartment in the LPS/D-GalN model, and found that *iNOS*, *Il6* or *Il10* were all upregulated at comparable levels in both ALF and hEnSC groups when compared to those of healthy group (Supplementary information, Fig. S7b), while we observed significant downregulations of *Cd16* and *Cd32* (two genes that encode Fc Gamma Receptor IIIa (FCGR3A) and Fc Gamma Receptor IIa (FCGR2A), respectively and are known to be important for the activation of neutrophils) in the hEnSC group, which indicates that neutrophils generally remained inflammatory with some changes upon hEnSC transplantation.^61^ These findings clearly demonstrated that the activation state of macrophages/Kupffer cells and the general hepatic immune micromilieu were skewed towards an anti-inflammatory state after hEnSC transplantation and that IFN signaling was significantly downregulated in liver macrophage populations. It would be interesting to interrogate whether or how hEnSCs or their derivatives were efferocytosed by macrophages/ Kupffer cells which might have a major effect on the latter. It would also be important to extend our research to the effects of hEnSC treatment on other innate and adaptive immune cell types, such as dendritic cells, NKT cells, innate lymphoid cells, Mucosa-associated invariant (MAIT) T cells and nonclassic T cells, as well as other hepatic lineages, including hepatic stellate cells and liver sinusoidal endothelial cells.

Type I IFNs and IFNγ are key regulators of immunity and inflammation, which induce overlapping ‘interferon signatures’ of canonical ISGs that encode molecules important for antigen presentation, inflammation, antiviral responses and autoimmunity.^62^ As the prototypic macrophage-activating factor, IFNγ is expressed by natural killer (NK) cells, Th1 cells, CD8^+^ cytotoxic T cells (CTLs) and innate lymphoid cells (ILCs) in response to stimulation from antigen receptors and inflammatory cytokines. It plays essential roles in macrophage polarization and priming, which also functions to promote the differentiation of Th1 cells and suppress responses mediated by Th2 and Th17 cells. The ex vivo and in vivo data in this study clearly demonstrated that hEnSC transplantation led to marked suppression of IFNγ signaling in the liver MoMF/Monocyte/Kupffer cell population from the D-GalN-induced ALF animals, as evidenced by the consistent downregulation of ISGs at both bulk and single-cell resolutions (Figs. 3b, 4c; Supplementary information, Figs. S8, S10, S11). Additionally, our data suggested that direct cell–cell contact or paracrine effects might be required for efficient suppression of IFNγ signaling in MoMFs/Kupffer cells, and pinpointed the unique importance of hEnSCs’ ability to target MoMFs/Kupffer cells, as we demonstrated both in vitro and in vivo (Fig. 4a, b; Supplementary information, Videos S2, S3). The marked reduction of Th1 cells (Fig. 2f) is likely a direct consequence of IFNγ inhibition. Moreover, as for iNKT cells, one of the dominant sources of IFNγ in rodent livers, we observed that the percentage of CD45^+^CD1d^+^IFNγ^+^ iNKT cells significantly decreased in hEnSC-transplanted group, when compared to that of ALF group (Supplementary information, Fig. S7a). Finally, it is noteworthy that precautions must be taken if the strategy of blocking IFNγ signaling were to be used for ALF treatment, and the appropriate antibiotics with minimal liver toxicity might be prescribed at the same time, as we observed that blocking IFNγ compromised the ability of MoMFs/ Kupffer cells to eliminate bacteria (data not shown).

Macrophages polarized by IFNγ are hypersensitive to various inflammatory stimuli, including TNFs, TLR ligands and LPS, and are prone to super-induction of inflammatory cytokines and canonical NF-κB target genes, a phenomenon known as “priming”, which epigenetically prepared inflammatory genes for subsequent challenge.^44^ Although not covered in this study, it would be important in future work to monitor the changes in the epigenomic profile of MoMFs/Kupffer cells after hEnSC treatment, with a focus on the assembly and disassembly of preexisting or latent enhancers, as it will provide insight into how hEnSCs function to reverse IFNγ-induced macrophage polarization and priming and how the epigenomic changes would affect consequent tissue repair. IFNγ-induced epigenomic remodeling also mediates gene-specific refractoriness to anti-inflammatory factors, including IL10, IL4, IL13 and glucorticoids, in primed macrophages.^63–67^ In line with this, we observed significant upregulation of anti-inflammatory cytokines (*Il10* and *Il12*) as well as the downregulation of proinflammatory factors (*Il1β, Gm-csf*) in the hEnSC-transplanted liver (Supplementary information, Fig. S13), most likely due to the inhibition of IFNγ signaling. It appears that the inhibition of IFNγ signaling and the activation of IL10 signaling synergized to reprogram the macrophage subpopulations towards an immunoregulatory state.

In an attempt to identify the key hEnSC-expressing regulator that functions to alleviate ALF, we found that CST1, a type 2 cystatin family member unique to primates,^68^ was able to mediate the suppression of IFNγ signaling directly. We revealed physical interactions between CST1 and the IFNGRs (Fig. 6a), which directly interfered with the ligand–receptor binding (Fig. 6b–d). These findings demonstrate a novel mechanism underpinning the intricate interplay between cystatin proteins and IFNγ signaling.

**Fig. 7:**
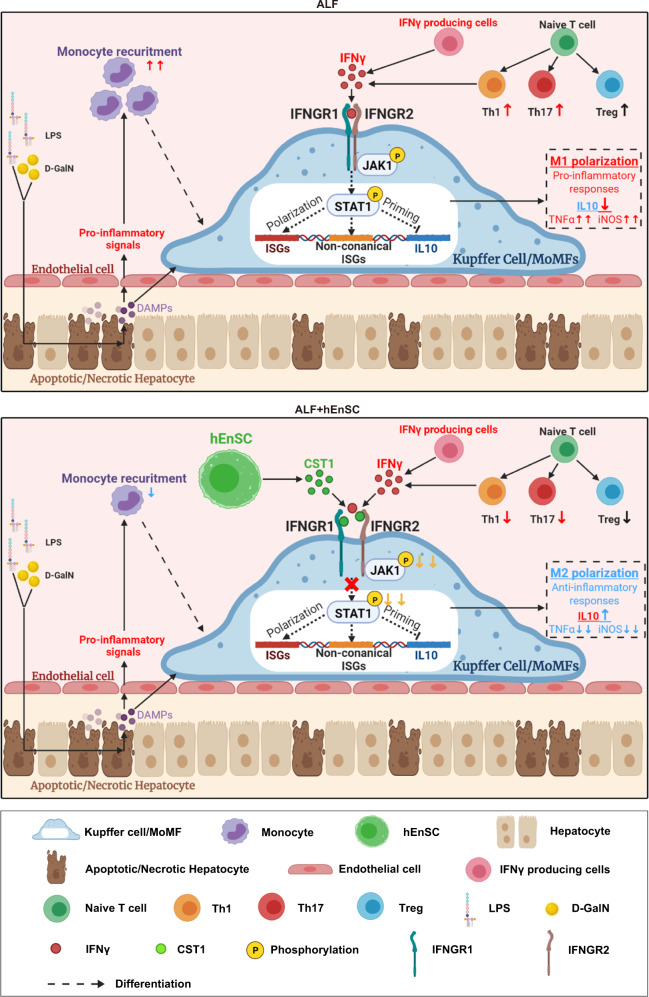
Schematic illustration of proposed working model of how hEnSC transplantation affects liver microenvironments. hEnSCs attract MoMFs/Kupffer cells in ALF livers and secrete CST1 in a paracrine fashion to suppress the IFN-induced pro-inflammatory macrophage polarization, and to promote the reprogramming of MoMFs/Kupffer cells into anti-inflammatory phenotypes by IL10, which finely tunes the immune microenvironments to accelerate the resolution of inflammation.

Through epistatic analyses after knockdown, overexpression and antibody administration, we clearly demonstrate the key hepatoprotective role of hEnSC-derived CST1, which functions by inhibiting IFN signaling in MoMFs and Kupffer cells and thereby upregulating IL10 and modulating macrophage activation states (Fig. 5d, e). We also demonstrated that the intraportal injection of CST1 protein alone was able to alleviate inflammatory hepatic injuries at 1 mg/rat, which approximates the efficacy of hEnSC transplantation (Fig. 5f, g) and suggests that a 60 kg ALF patient might need 300 mg CST1 as far as the weight is only concerned. However, the viability of the clinical application of CST1 treatment will depend on rigorous safety and efficacy evaluations in the future. hEnSC transplantation might still have some advantages over CST1 treatment, considering the unique abilities of hEnSCs to home injured liver and to attract macrophage/Kupffer cells (Figs. 1c, d, 4a, b), which allows the precise local delivery of immunomodulatory signals. Finally, we propose a model in which hEnSCs attract MoMFs/Kupffer cells in the ALF liver and secrete CST1 in a paracrine fashion to suppress IFN-induced proinflammatory macrophage polarization and to promote the reprogramming of MoMFs/Kupffer cells into an anti-inflammatory phenotype by IL10, which finely tunes the immune microenvironment to accelerate the resolution of inflammation (Fig. 7).

In summary, with rodent and swine models, we propose hEnSC transplantation as a novel potential cell therapeutic treatment for inflammation-related ALF patients. In this study, the phenotypic and functional conversion of monocytes/MoMFs/Kupffer cells in the context of ALF and its cure was revealed for the first time at single-cell resolution, providing insights and resources for future studies concerning immunomodulatory events in tissue repair and regeneration. The mechanism underpinning the immunoregulatory effect of stem cell transplantation, particularly CST1-based intervention, will shed light on the development of new therapeutic strategies for curing liver diseases or other diseases that involve IFN over-activation. Current efforts are focused on the optimization of hEnSC transplantation and CST1 per se as candidate medications for clinical trials.

## Materials and methods

### Establishment, maintenance and expansion of hEnSC lines

H9 and H1 hESC lines were provided by Core Facility for Stem Cell Research, CAS Center for Excellence in Molecular Cell Science, China. hEnSC lines were generated from H9 and H1 hESCs and were maintained in serum-free conditions as described previously.^16^

hEnSCs for transplantation were harvested on day 5 of culture and dissociated into single cells with 0.25% Trypsin-EDTA (Gibco, Cat# 25200056) for 5 min at 37 °C.

### Derivation, maintenance and preparation of control cells for transplantation

The establishment, maintenance or isolation of H9-EGFP-hESCs, H9-EGFP-hEnSCs, human umbilical cord-derived mesenchymal stem cells (hUC-MSCs) and rodent primary hepatocytes are described in Supplementary information, Data S1. All cell lines were tested routinely and were negative for mycoplasma.

### ALF animal models and in vivo procedures

WISTAR rats (6–8 weeks old, male, 180–200 g), C57BL/6 mice (8–10 weeks old, 18–20 g, male) and Chinese Bama miniature pigs (4–5 months, either sex, ~25 kg) were purchased from the Laboratory Animal Center of the Affiliated Drum Tower Hospital of Nanjing University Medical School. Animal procedures were performed according to institutional and national guidelines and approved by the Animal Care Ethics Committees of Nanjing University and Nanjing Drum Tower Hospital and CAS Center for Excellence in Molecular Cell Science.

The induction of ALF in rodents and minipigs and the detailed in vivo procedures are described in Supplementary information, Data S1.

### Live imaging for tracing transplanted cells in vivo

hEnSCs or hMSCs were incubated with 50 μM DiR for 20 min at 37 °C according to the manufacturer’s protocol (Fanbo Biochemicals, Beijing, China) and intraportally transplanted in 1 mL PBS. Image acquisition was performed using IVIS® Spectrum In vivo Imaging System (PerkinElmer). Laser excitation wavelengths of 748 nm were used for fluorescence detection. More details are described in Supplementary information, Data S1.

### Isolation of rat liver monocyte/MoMFs/Kupffer cells and lymphocytes

The isolation method involves in situ perfusion followed by purification on density gradient,^69^ and is described in Supplementary information, Data S1.

### RNA extraction and qRT-PCR

The reverse transcription and qRT-PCR reactions were performed as reported previously,^16^ and described in Supplementary information, Data S1. Primers were listed in Supplementary information, Table S1.

Human cell numbers in rat tissues (human cell numbers/10^6^ rat cells) were estimated by qRT-PCR that was designed to quantify the transcripts of human specific *Alu* sequence based on the standard curve created by mixing human and rat cells at ratios of 1:100, 1:1000, 1:10^4^, 1:10^5^ and 1:10^6^.

### Cytokine array

The media were collected and pooled from hEnSCs or hUC-MSCs that were cultured for 5 days, and were assayed for inflammation-related secreted proteins with RayBio® C-Series Human Inflammation Array C1 (RayBiotech, Inc., Guangzhou, China).

### Flow cytometry and antibodies

The staining of intracellular proteins followed the published protocols.^16^ The details and the antibodies used are described in Supplementary information, Data S1.

### In vitro coculture of hEnSCs and liver monocytes/MoMFs/Kupffer cells

1 × 10^5^ liver monocytes/MoMFs/Kupffer cells were isolated from rat livers 24 h post D-GalN treatment, and were seeded with hEnSCs at a ratio of 1:1 in 12-well tissue culture dishes (Falcon, Cat# 353043). The cells were harvested for RNA extraction on day 3 of coculture.

In the transwell assay, hEnSCs were seeded at 1 × 10^5^ cells/well in the transwell (Costar, Cat# 3493), and the liver monocytes/MoMFs/Kupffer cells were seeded in 12-well tissue culture dishes at 1 × 10^5^ cells/well. Cells were cultured in RPMI-1640 supplemented with 10% FBS and 1% P/S. The liver monocytes/MoMFs/Kupffer cells were harvested for RNA extraction on day 3 of coculture.

### In vitro live imaging for monitoring the migration between hEnSCs and MoMFs/Kupffer cells

1 × 10^5^ MoMFs/Kupffer cells that were isolated from ALF rat livers and labeled with DiI-Ac-LDL or 1 × 10^5^ EGFP-hEnSCs were embedded separately in 20 μL cold Matrigel (CORNING, Cat# 354230, 4 °C) and transferred with tips onto a well of 24-well dish (Falcon) and let solidified at 37 °C for 5 min in incubator before the RPMI-1640 (10% FBS, 1% P/S) was added to the well. The gaps between the solidified Matrigel drops were kept less than 500 μm. Photos were acquired every 30 min with Operetta CLS High-Content Analysis System at 37 °C 5% CO_2_ for 24 h.

### Intravital microscopy to monitor in vivo interactions between hEnSCs and MoMFs/Kupffer cells

Mouse ALF was induced with intraperitoneal injection of D-GalN 24 h ahead of cell transplantation. Surgical preparation for liver intravital imaging was performed as described.^70^ 1 × 10^6^ EGFP-hEnSCs or H9 hESCs were injected into the portal vein before imaging. Image acquisition was performed using an inverted Olympus FV3000 confocal microscope with a 20×/0.75 UPLANSAPO objective lens. Data analysis was conducted using ImageJ (FIJI). More details can be found in Supplementary information, Data S1.

### scRNA-seq and data processing

scRNA-seq was performed according to the 10× genomics sequencing protocol as reported previously,^71^ and the details and data processing are described in the Supplementary information, Data S1. Three samples (healthy, PBS-treated ALF and hEnSC-transplanted ALF) were sequenced, and for each sample, the adherent cells containing rat liver monocyte/MoMF/Kupffer cell populations were isolated from 3 rats with identical treatments, and mixed before cDNA library construction. The data processing was performed on R package Seurat 2.3.

### Plasmid construction, gene expression, protein purification and biochemical analyses

Please refer to Supplementary information, Data S1 for details.

### Data analysis

All data are presented as means ± SD. Log-rank (Mantel-Cox) test was applied for significance analysis between survival curves. For most statistic evaluation, paired or unpaired two-tailed Student’s *t*-tests were performed when two groups of samples were compared. One-way or two-way ANOVA with Tukey’s or Sidak’s tests were performed when multiple groups were compared. All the *P* values were calculated using GraphPad PRISM 5, with the statistical significance defined as *P* < 0.05. See statistical details for each experiment in the figure legends.

## Supplementary information

Supplementary information, Fig. S1

Supplementary information, Fig. S2

Supplementary information, Fig. S3

Supplementary information, Fig. S4

Supplementary information, Fig. S5

Supplementary information, Fig. S6

Supplementary information, Fig. S7

Supplementary information, Fig. S8

Supplementary information, Fig. S9

Supplementary information, Fig. S10

Supplementary information, Fig. S11

Supplementary information, Fig. S12

Supplementary information, Fig. S13

Supplementary information, Fig. S14

Supplementary Information, Table S1

Supplemantary information, Data S1 Materials and methods

Supplementary video legends

Video S1

Video S2

Video S3

Video S4

## Data Availability

The scRNA-seq data have been submitted to the National Omics Data Encyclopedia (NODE). The following link has been created to permit the review of record OEP000196, while ensuring that it remains private: https://www.biosino.org/node/project/detail/OEP000196. Further information and requests for resources and reagents should be directed to and will be fulfilled by the lead contact, Xin Cheng (xcheng@sibcb.ac.cn).
