# Supplementary material for: Human endoderm stem cells reverse inflammation-related acute liver failure through cystatin SN-mediated inhibition of interferon signaling
Source: Cell Res. 2023 Jan 20;33(2):147–64. doi: 10.1038/s41422-022-00760-5 (PMC9892047; doi:10.1038/s41422-022-00760-5)
Supplement: Supplementary file 2 — Supplementary information, Fig. S2 [file 41422_2022_760_MOESM2_ESM.pdf]

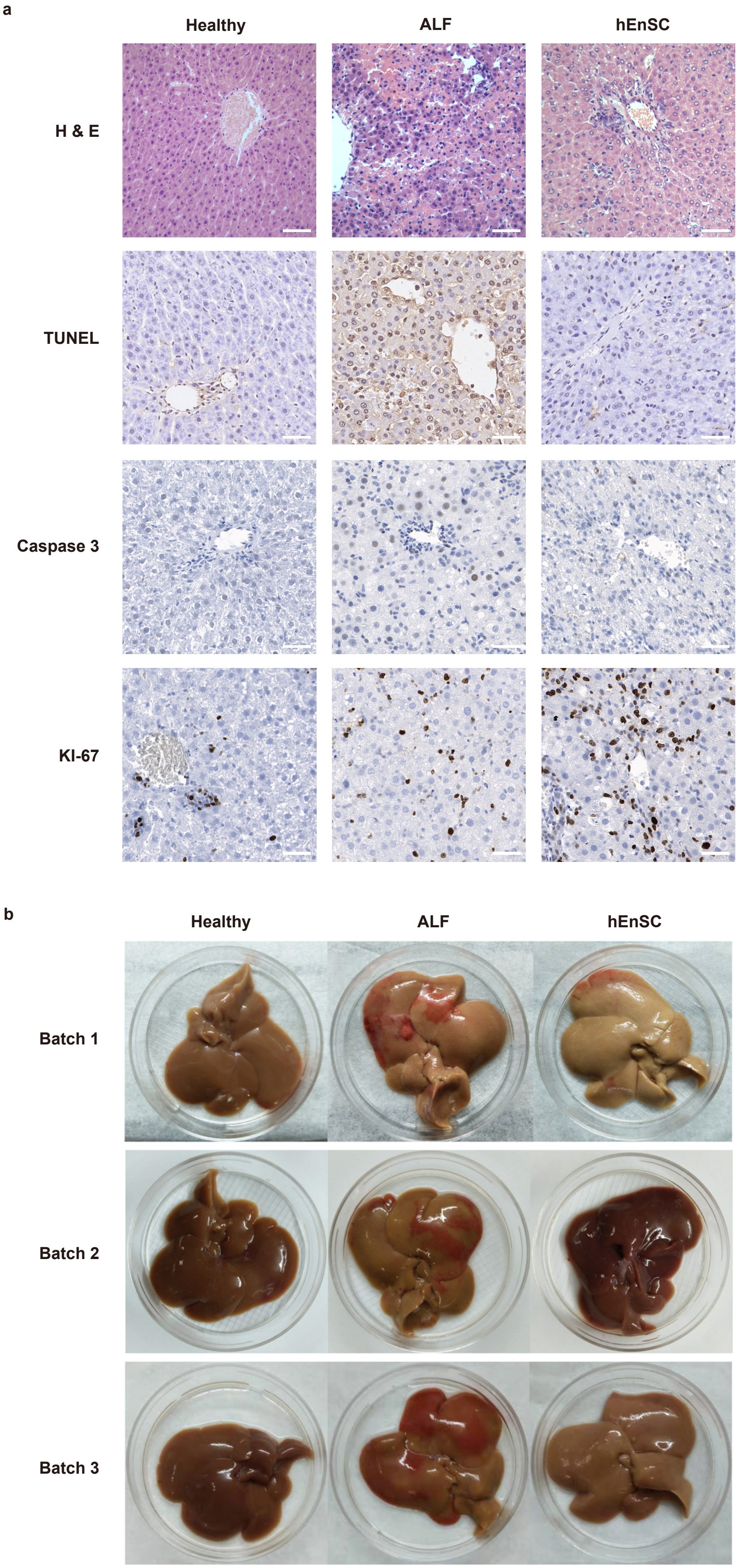

Fig. S2 Histological and Pathological Analyses of the Liver Samples Isolated from Healthy, ALF and hEnSC-Transplanted Animals in D-GalN induced rat ALF model. a Images of H & E, and TUNEL staining, Caspase 3 and KI-67 staining of rat liver sections. Liver samples were collected at day 3 post drug administration. Scale bar: 50 μm. **b** Representative images displaying the morphology of whole livers dissected from rats of different groups. Liver samples were collected at day 3 post D-GalN administration and perfused with HBSS. Healthy: healthy rats; ALF: PBS-treated ALF rats; hEnSC: hEnSC-transplanted ALF rats.
