# Supplementary material for: Human endoderm stem cells reverse inflammation-related acute liver failure through cystatin SN-mediated inhibition of interferon signaling
Source: Cell Res. 2023 Jan 20;33(2):147–64. doi: 10.1038/s41422-022-00760-5 (PMC9892047; doi:10.1038/s41422-022-00760-5)
Supplement: Supplementary file 15 — Supplementary Information, Table S1 [file 41422_2022_760_MOESM15_ESM.pdf]

Table S1. qRT-PCR Primer List.

Gene	Sequences (Forward, 5' to 3')	Sequences (Reverse, 5' to 3')
TBP	TTGCTGAGAAGAGTGTGCTGGAGATG	CGTAAGGTGGCAGGCTGTTGTT
CST1	TCCCGGGTGGCATCTATAAC	CGGACGTCTGTAGTAGTCATCT
TGFβ1	CAAGCAGAGTACACACAGCAT	TTAACTTGAGCCTCAGCAGAC
CCL2	CCATTGTGGCCAAGGAGAT	CAAGTCTTCGGAGTTTGGGT
CST3	GAACTACTTCTTGGACGTGGAG	GATGTGGCTGGTCATGGAAG
CSTB	CCTGCGAGTGTTCCAATCT	GCTCATCATGCTTGGCTTTG
HLA-A	AAGGATTACATCGCCCTGAAC	ATCCAGGTAGGCTCTCAACT
HLA-B	GCTTCATCTCAGTGGGCTAC	GTGTTCCGGTCCCAATACTC
HLA-C	GATGGAACCTTCCAGAAGTGG	CTGCATATGGCACGTGTATCTC
HLA-DRA	CCCAACGTCCTCATCTGTTT	ACTGTCTCTGACACTCCTGT
HLA-DRB1	GAGTACTGGAACAGCCAGAAG	CGTAGTTGTGTCTGCAGTAGG
Tbp	GACTCCTGGAATTCCCATCTT	CTGCTGTCTTTGTTGCTCTTC
Tnfα	GCAGATGGGCTGTACCTTATC	GAAATGGCAAATCGGCTGAC
iNos	AGGAACCTACCAGCTCACT	ACTGAATCCTGCCGATGC
Il6	CACAGAGCAGAGAATGGACTAC	CACAGTCCCAACCAGATGAT
Il10	CAGTGGCAAACCATCACTTC	CTTTCCCAGAGTCTCCTGTATC
Cxcl10	TGAGCTGCATTCCAATCCC	GAGAACGACAGCAGCACTT
Mx1	CCTGAAGAAGGGCTACATGAT	TGAGGGTGTTCCTTGAAGAAG
Irf7	CCAGATGCGTGTTCCTGTAT	GTAGTGCAGCTGCTTCTGAT
Isg15	GACACATCAGGTCAGTTCCTAC	CCTTCACAGCTTACCCAGATAG
Ifit2	GTGTGAAGACAGAGCCATCTAC	CATCTCTAGAAAGCCGCCTTAG
Mx2	TGCATTGACCTCATCGACTC	CACAGAGCTCTTCCCTGAAC
Eif2ak2	ATGGCCAGTGATACACCAG	CTTCTGTCGTGTGGAGGT
Sp100	AGATTCGTATAGAGCCCTGGT	GAACACTTCACAGAGGACCTTC
Ifit3	GAATACAGGAGAGGCTGACAAG	CTACTTCCGGGAAATCGATGAG
Ifitm3	CTGCCAAATGCCTGAACATC	CTGGAGACGAGGAGCATTAAG
G-csf	GCAGGCTCTATCGGGTATTTC	CTGGAAGGCAGAAGTGAAGG
Mip-2	CTGTCAATGCCTGAAGACCC	TGGCTATGACTTCTGTCTGGG
Il1a	AGCACCTTACACCTACCAGAGTG	TCCCGACGAGTAGGCATACA
Il1β	AAGAAGAGCCCATCCTCTGTG	TGTTCATCTCGGAGCCTGTAG
Il2	AGATGAACTTGGACCTCTGCG	CAGAAAGTCCACCACAGTTGC
Il4	TGAACGAGGTCACAGGAGAAG	TTGGAAGCCCTACAGACGAG
Il5	GCAATGAGACGATGAGGCTTC	CCCCACGGACAGTTTGATTC
Il13	GGCAGCATGGTATGGAGTGT	TCTGGGTCCTGTAGATGGCA
Il12p70	GAGCACTCCCCATTCCTACT	TCTGGTTACACCCCTCCTCT
Gm-csf	CAAAGAAGCCCTGAACCTCC	CCGTAGACCCTGCTCGAATA
Il17a	CTCAGACTACCTCAACCGTTCC	ATGTGGTGGTCCAGCTTTCC
Human Alu sequence	GACCATCCCGGCTAAAACG	CGGGTTCACGCCATTCTC
