# Supplementary material for: Human endoderm stem cells reverse inflammation-related acute liver failure through cystatin SN-mediated inhibition of interferon signaling
Source: Cell Res. 2023 Jan 20;33(2):147–64. doi: 10.1038/s41422-022-00760-5 (PMC9892047; doi:10.1038/s41422-022-00760-5)
Supplement: Supplementary file 16 — Supplemantary information, Data S1 Materials and methods [file 41422_2022_760_MOESM16_ESM.pdf]

Supplementary materials and methods

Derivation, maintenance and preparation of control cells for transplantation

H9 hESCs that carry lentiviral EGFP were maintained under feeder-free conditions on growth factor-reduced Matrigel (CORNING, Cat# 354230) (1:6 diluted in IMDM) (Thermo Fisher, Cat# 12200036) and fed daily with mTeSR medium (STEMCELL, Cat# 85850). Accutase (STEMCELL, Cat# 07920) was used for dissociation of hESCs into single cells, and Rock inhibitor Y-27632 (10μM, Selleck, Cat# S1049) was supplemented for the first 24 h after passage. H9 hESCs (passages 65 and 72) were harvested at conflucency of 70% and were dissociated into single cells with TrypLE™ Express (Gibco, Cat# 12605010).

Human umbilical cord-derived mesenchymal stem cells (UC-MSCs) were from Clinical Stem Cell Center of Nanjing Drum Tower Hospital. The purification and identification procedures were described previously.[1, 2] The UC-MSCs were cultured in DMEM with low glucose containing 10% fetal bovine serum (Gibco, Cat# 10099141) plus 1% Penicillin-Streptomycin (Cellgro, Cat# R30-002-CI). For transplantation, human UC-MSCs were expanded till passage 4 after isolation and purification.

Rat primary hepatocytes were freshly isolated and harvested by a two-step in situ collagenase perfusion procedure previously described with modifications.[3]

All cell lines were tested routinely and were negative for mycoplasma.

ALF animal models and *in vivo* procedures

WISTAR rats (6-8 weeks old, male, 180-200 g), C57BL/6 mice (8-10 weeks old, 18-20g, male) and Chinese Bama miniature pigs (4-5 months, either sex, ~25 kg) were purchased from the

Laboratory Animal Center of the Affiliated Drum Tower Hospital of Nanjing University Medical School. All animals were housed in individually ventilated cages in specific pathogen-free animal facility with temperature and light controlled (12-h light/dark cycle). All animals were anesthetized with isoflurane.

The induction of ALF in rats was achieved by an intraperitoneal injection of D-galactosamine hydrochloride (D-GalN) (Sigma, Cat# G0500) at 1.0 g/kg for D-GalN-induced model, or by simultaneous intraperitoneal injections of D-GalN (800 mg/kg) and LPS (Sigma, Cat# L2880; 20 μg/kg) for D-GalN + LPS induced model. One day post D-GalN treatment, $1 x 10^7$ cells were resuspended in 1 mL PBS and intraportally injected into each rat over a period of 5 minutes. The ALF swine model was induced by one intravenous administration of D-GalN at dosage of 0.4 g/kg. $5 x 10^8$ cells hEnSCs were intraportally infused over a period of 10 minutes, 1 day post D-GalN administration. The APAP-induced ALF model was established by orally administering APAP (MCE, Cat# HY-66005) in C57BL/6 mice at 300 mg/kg, single dose.

The sera of rodents and pigs were collected from the tail vein under anaesthesia at every day until the completion of the study or the death of animals (7 days after D-GalN or APAP treatments). As for the swine model, as 8 out of 10 animals in the control group died within 48 hours, we were hence not able to collect sera of these animals at days 2 or 3, and therefore the liver injury parameters of the control are from the 2 animals survived more than 2 days. The liver tissues were excised after euthanasia and fixed with 4% paraformaldehyde and processed for histological and immunohistochemical analyses.

Live imaging for tracing transplanted cells *in vivo*

Before transplantation, hEnSCs or hMSCs were incubated with 50 μmol/L DiR for 20 min at 37 °C according to the manufacturer's protocol (Fanbo Biochemicals, Beijing, China). The DiR-labelled hEnSCs or MSCs were centrifuged at 453 g for 5 min and the cells were resuspended in PBS. This procedure was repeated twice to ensure complete removal of any residual dye. The DiR-labeled cells were intraportally transplanted in 1 mL PBS. Image acquisition was performed using IVIS® Spectrum In Vivo Imaging System (PerkinElmer). Laser excitation wavelengths of 748 nm were used for fluorescence detection.

Isolation of rat liver Monocyte/MoMFs/Kupffer cells and lymphocytes

Rats were anesthetized with intraperitoneal injection of 10% Chloral hydrate (300 μl/100 g). Hepatic portal vein was exposed by carefully moving the viscera to the right outside of the abdominal cavity, and an 18-gauge angiocath was inserted into portal vein. The inferior vena cava was cut and the perfusion was started immediately. For each rat, perfusion was performed with prewarmed HBSS (37℃, without $Ca2^{+}$ and $Mg2^{+}$) at low rate (10 mL/min) for 10 min, and repeated for another 10 min. Then the liver was excised and cut into segments with scissors. Twenty mL 0.05% collagenase IV (Gibco, Cat# 17104019) in RPMI-1640 (supplemented with 5% Fetal Bovine Serum (Gibco) and 1% Penicillin-Streptomycin (Cellgro) was used to resuspend the liver slurry, and the samples were digested in 37℃ water bath shaker for 1 hour. The homogenate was then filtered through a 70 μm sterile cell strainer, and separated with density gradient centrifugation at 300 g, 10 min, 4℃. With the supernatants discarded, the cell pellets were suspended with 20 ml 40% Percoll (4℃) and carefully layered onto 20 mL 70% Percoll (4℃) in a 50 mL centrifuge tube, centrifuged at 1260 g for 15 min, 4℃, brake off. The

interface of the layers was collected and added to another 50 mL tube with cold HBSS, and centrifuged at 1260 g for 10 min, 4℃. The pellet was suspended with 6 mL ice-cold HBSS and layered onto 6 ml Lymphoprep™ (STEMCELL, Cat# 07801) in a 15 mL tube, and centrifuged at 450 g for 10 min, 4℃, brake off. The interface of layers was collected and centrifuged again at 1260 g, 10 min, 4℃. Cell pellets were suspended in RPMI-1640 (Gibco, Cat# 72400120) supplemented with 10% FBS and 1% P/S and seeded into 6-well tissue-culture dishes at the density of 0.25×10^6/well. After 15 min incubation at 37℃, the nonadherent cells were liver monocyte/macrophage-depleted lymphocytes, the adherent cells were mainly liver monocytes/MoMFs/Kupffer cells. Nonadherent lymphocytes were collected and centrifuged at 1260 g, 10 min, suspended in PBS for FACS staining immediately. Adherent monocytes/MoMFs/Kupffer cells were washed with RPMI-1640 twice to remove the nonadherent cells, then cultured in RPMI-1640 supplemented with 10% FBS and 1% P/S, 37℃ for 1 hour before subsequent experiments.

RNA extraction and quantitative real-time PCR

The reverse transcription and qRT-PCR reactions were performed as reported previously.[4] The RNAs was prepared with an RNA kit (TIANGEN, Cat# DP420) reverse-transcribed into cDNAs using random hexamers and oligo (dT) primers with GoScript™ Reverse Transcription System (Promega, Cat# A5001). The qRT-PCR reactions were performed using an ABI Q6 (Life) system and SYBR Green Master Mix (Roche, Cat# 4913850001). For gene detections, the expression levels were normalized to the housekeeping gene human *TBP or rat Tbp*. Human and Rat genomic DNAs were extracted using TIANamp Genomic DNA Kit (TIANGEN, Cat#

dp304) and used to build absolute quantification standard curve. Primers were listed in Supplementary information, Table S1.

Antibodies

Antibodies used for flow cytometry were purchased from Miltenyi Biotec (PE anti-rat CD68, Cat# 130-103-363; Anti-rat CD4, VioBlue, Cat# 130-107-666), Biolegend (PE anti-rat CD86, Cat# 374205; Anti-rat CD25, Alexa Fluor® 488, Cat# 202107; Alexa Fluor® 647 anti-mouse/rat/human FOXP3, Cat# 320014; PE anti-rat IFN-gamma, Cat# 507806; Purified Mouse IgG1, κ Isotype Ctrl, Cat# 401402; Purified rat IgG1, kappa isotype Ctrl, Cat# 400402), Biorad (Anti rat CD163, Cat# MCA342R), Thermo Fisher Scientific (PE anti IL-17A, Cat# 12-7177-81; Streptavidin PE-Cy7, Cat# 25-4317-82; Anti-human CXCR4 (CD184), R-PE, Cat# MHCXCR404; Anti-human c-Kit (CD117), APC, Cat# CD11705), R & D Systems (Anti-human EOMES MAb, Cat# MAB6166; Anti-human Sox17 Polyclonal antibody, Biotin, Cat# BAF1924; Anti-human FOXA1, Cat# MAB6778; Normal goat IgG Biotinylated, Cat# BAF108), BD Biosciences (Streptavidin-Phycoerythrin, Cat# 554061; Streptavidin-Allophycocyanin, Cat# 554067), Santa Cruz Biotechbology (Normal mouse IgG2a, Cat# sc-3878; Normal mouse IgG2b, Cat# sc-3879).

Antibodies used for co-immunoprecipitations and western blots were purchased from Abcam (Anti-human Cystatin SN, Cat# ab68329), ABclonal (Anti-rat IFNGR1, Cat# A5748; Anti-rat IFNGR2, Cat# A7558; Anti-rat CST3, Cat# A1561; Anti-His tag, Cat# ae068), Proteintech (Anti-CST1, Cat# 16025-1-AP) Beyotime (Anti-IFNG, Cat# AF7173), Cell Signaling (Anti-rabbit IgG, HRP-linked, Cat# 7074; Anti-mouse IgG, HRP-linked, Cat# 7076).

Antibodies used for phosphorylated protein western blots were purchased from Cell Signaling (Anti-Phospho-Stat1, Cat# 9167; Anti-Phospho-Stat3, Cat# 9145; Anti-Phospho-Stat6, Cat# 9361; Anti-Phospho-Jak1, Cat# 3331; Anti-Phospho-Jak2, Cat# 3771; Anti-Phospho-Jak3, Cat# 5031), Abcam (Anti-STAT1, Cat# ab281999; Anti-STAT2, Cat# ab32367; Anti-STAT3, Cat# ab68153; Anti-STAT6, Cat# ab32520; Anti-JAK1, Cat# ab133666; Anti-JAK3, Cat# ab203611), ABclonal (Anti-Phospho-STAT2, Cat# AP0284; Anti-Phospho-Tyk2, Cat# AP0543), Beyotime (Anti-JAK2, Cat# AF1489; Anti-TYK2, Cat# AF8280), Yeasen (Anti-GAPDH, HRP conjugated, Cat# 30203ES10).

Flow cytometry

For staining of surface markers, the cells were incubated with antibodies in PBS with 0.1% BSA for 30 min on ice, and then washed by staining buffer for 3 times. The staining of intracellular proteins followed the published protocols[4]. Briefly, the cells were fixed with 1.6% paraformaldehyde in PBS at room temperature for 20 min, and washed with the 1X Intracellular Staining Permeabilization Wash Buffer (BioLegend, Cat# 421002), and then stained with first and second antibodies in 1X Intracellular Staining Permeabilization Wash Buffer. For co-staining of extracellular and intracellular markers, the cell surface markers (CD4, CD25, CD1d, and CD45) were stained before cell fixation. True-Nuclear™ Transcription Factor Buffer Set (Biolegend, Cat# 424401) was specialized for FOXP3 staining. Cell analyses were performed on BD LSRFortessa™ platform.

H&E and TUNEL staining of liver sections

After euthanasia liver tissues were excised, fixed in 4% paraformaldehyde for 24 hours. Three fragments, each measuring less than 3 mm in thickness, were obtained from each liver. Sections were stained with hematoxylin and eosin (Beyotime Biotechnology, Shanghai, China), or TUNEL kit (Roche, Cat# 11772465001) for pathological assessment.

Measurement of liver functional parameter (ALT, AST, NH_3, PT and CRP)

The sera were collected for analyses under anaesthesia from the tail vein for rats and mice, or from the ear vein of abdominal aorta for pigs, every 24 hours after D-GalN or APAP treatment, except for day 1 when the sera were collected 6 hours post-transplantation (i.e. 30 hours post D-GalN or APAP treatment). The Sera collected at day 1 were 6 hours post transplantation in D-GalN induced ALF rat and swine models. The serum levels of ALT, AST, NH_3, PT and CRP were determined with an automated biochemical analyzer iMagic-M7 (Mindray, Shenzhen, China).

Intravital microscopy to monitor *in vivo* interactions between hEnSCs and MoMFs/Kupffer cells

Mouse ALF was induced with intraperitoneal injection of D-GalN (750 mg/kg) 24 hours ahead of cell transplantation. Before cell transplantation, mice were anesthetized with Avertin (20 mL/kg i.p.) and a catheter was inserted via tail vein for delivery of the fluorescently labeled antibodies and anesthetic. Surgical preparation for liver intravital imaging was performed as described.[5] In brief, the abdominal cavity was exposed by removing the skin and muscles. The mouse was then placed on a heated stage (37℃) and the largest lobe of the liver was positioned

onto a coverslip; a small piece of sterile laboratory wipes was moisturized with saline and covered on the liver to keep liver moist and stable. Kupffer cells in liver sinusoid were visualized by intravenous infusion of 5 μg PE-conjugated anti-F4/80 (Biolegend, Cat# 123110). 1×10^6 EGFP-hEnSCs or H9 EGFP-hESCs were injected into the portal vein before imaging. Image acquisition was performed using an inverted Olympus FV3000 confocal microscope with a 20x/0.75 UPLANSAPO objective lens. Laser excitation wavelengths of 488nm and 561nm and 2 HyD spectral detectors were used for fluorescence detection. Two fields of interest were acquired simultaneously for each mouse, with a scan-field dimension of 320 μm x 320 μm (800 x 800 pixels) and 2 μm Z-step size for 6 μm, taken every 90 seconds for 1-3 hours. Data analysis was conducted using ImageJ (FIJI).

Single-cell RNA sequencing and data processing

Single-cell RNA sequencing was performed according to the 10x genomics sequencing protocol as reported previously.[6] Three samples (healthy, PBS-treated ALF and hEnSC-transplanted ALF) were sequenced, and for each sample, the adherent cells containing rat liver monocyte/MoMF/Kupffer cell populations were isolated from 3 rats with identical treatments, and mixed before cDNA library construction. The 10x Genomics libraries were sequenced as 150-bp paired-end reads on the Illumina HiSeq 4000 platform. Raw files were processed with Cell Ranger 2.0.2 using the default parameters. Reads were mapped to the Rattus norvegicus reference genomes (Rnor 5.0) provided by 10x Genomics (https://support.10xgenomics.com/single-cell-gene-expression/software/downloads/latest).

Cell filtering was performed with Cell Ranger using the default settings. A total of 12177

cells (healthy, 3529 cells; untreated ALF, 4828 cells; hEnSC-transplanted ALF, 3820 cells) were retained after filtration. We further selected for monocyte/macrophage/Kupffer cells by removing the *Cd68*- cells, low-expressing cells (number of detected genes < 500) and outliers, which resulted in 10927 cells (healthy, 3041 cells; untreated ALF, 4274 cells; hEnSC-transplanted ALF, 3612 cells) retained for the subsequent analyses. To eliminate batch effects among different cell samples and demarcate the population heterogeneities across the three groups, the sum of counts were normalized for each cell to 10000 and the data were further corrected based on house-keeping genes.[7] The data processing was performed on R package Seurat 2.3.

Plasmid construction, gene expression and protein purification

Rat IFNGR1, IFNGR2, IFNAR1 extracellular segment sequences and human CST1 structure information were obtained from UniProt. IFN receptor extracellular segment sequences and mutated CST1 sequences were synthesized by GenScript and inserted between NcoI and XhoI sites of pET28a vector with a C-terminal His tag. These plasmids were transfected into BL21 Competent E.Coli (TIANGEN), gene expressions were induced with 200μM isopropyl β-D-1-thiogalactopyranoside (IPTG) when the initial E.Coli cell culture reached an absorbance at 600nm of 0.6, then cultured at 16℃ overnight. Protein purification was carried out by affinity chromatography on Ni-NTA Superflow according to the manufacturer’s protocol (QIAGEN).

Co-immunoprecipitation and western blot

Co-immunoprecipitations were carried out by using Pierce™ Classic Magnetic Co-

Immunoprecipitation (Co-IP) Kit, following the manufacturer's protocol (Thermo). Monocyte/MoMF/Kupffer cells used for the Co-IPs were isolated from ALF rat livers, and lysed using Co-IP lysis buffer described in the protocol. For the interaction between CST1 and IFNGR1 or 2, 1 mg Monocyte/MoMF/Kupffer cell lysate was used for each Co-IP sample, 3ug CST1 and 2ug anti-CST1 or rat IgG were added in the Co-IP system. In the Competition Co-IP assay, 1 mg cell lysate adding 1ug IFNG was used for each sample, and 2ug anti-IFNG used as Co-IP antibody, with or without adding 3ug CST1 to interrupt IFNG-IFNGR1 or IFNG-IFNGR2 interactions. 3ug CST1, 1ug IFNGR1 or 2 and 2ug anti-CST1 (or Rat IgG) was used for each sample in the Co-IP demonstrating the direct interactions between CST1 and IFNGR1 or 2. Similarly, when extracellular segments of IFN receptors were used in the Co-IP, 3ug IFNGR1 / IFNGR2 / IFNAR1 extracellular segment protein, 3ug CST1 and 2ug anti-CST1 was used for the Co-IP. When CST1 mutants were used, 3ug CST1 mutants (or wildtype CST1), 1ug IFNGR1 and 2ug anti-IFNGR1 was used for each sample.

Co-IP samples and protein samples were detected by western blots. Samples were separated on Precast SDS-PAGEs (meilunbio) and transferred to methanol-activated polyvinylidene fluoride (PVDF) membranes, then blocked with 5% BSA in TBST for 1h at room temperature. When phosphorylated proteins were detected, SDS-PAGE separation should carried out at 4℃。Immunoblotting was performed using primary antibodies overnight at 4℃, after washing with TBST three times, the membranes were incubated with HRP-conjugated secondary antibodies for 2h at room temperature. Detection was perform using High sensitive ECL luminescence reagent (Sangon Biotech) on a MiniChemi™ analysis system.

References

1 Xu J, Wang D, Liu D *et al.* Allogeneic mesenchymal stem cell treatment alleviates experimental and clinical Sjogren syndrome. *Blood* 2012; **120**:3142-3151.

2 Wang D, Feng X, Lu L *et al.* A CD8 T cell/indoleamine 2,3-dioxygenase axis is required for mesenchymal stem cell suppression of human systemic lupus erythematosus. *Arthritis Rheumatol* 2014; **66**:2234-2245.

3 Gu J, Shi X, Chu X, Zhang Y, Ding Y. Contribution of bone marrow mesenchymal stem cells to porcine hepatocyte culture in vitro. *Biochem Cell Biol* 2009; **87**:595-604.

4 Cheng X, Ying L, Lu L *et al.* Self-renewing endodermal progenitor lines generated from human pluripotent stem cells. *Cell Stem Cell* 2012; **10**:371-384.

5 Wang J, Kubes P. A Reservoir of Mature Cavity Macrophages that Can Rapidly Invade Visceral Organs to Affect Tissue Repair. *Cell* 2016; **165**:668-678.

6 Picelli S, Faridani OR, Bjorklund AK, Winberg G, Sagasser S, Sandberg R. Full-length RNA-seq from single cells using Smart-seq2. *Nat Protoc* 2014; **9**:171-181.

7 Das RK, Banerjee S, Shapiro BH. Extensive sex- and/or hormone-dependent expression of rat housekeeping genes. *Endocr Res* 2013; 38:105-111.
