# Supplementary material for: Human endoderm stem cells reverse inflammation-related acute liver failure through cystatin SN-mediated inhibition of interferon signaling
Source: Cell Res. 2023 Jan 20;33(2):147–64. doi: 10.1038/s41422-022-00760-5 (PMC9892047; doi:10.1038/s41422-022-00760-5)
Supplement: Supplementary file 17 — Supplementary video legends [file 41422_2022_760_MOESM17_ESM.pdf]

Video S1. Video showing behavioral phenotypes of ALF rats with different treatments

Video S2. Video captured with in vitro live imaging system to show the migration and attraction between EGFP-hEnSCs and DiI-Ac-LDL-labelled MoMFs / Kupffer cells
Macrophages were labelled with DiI-Ac-LDL before the experiment. EGFP-hEnSCs and DiI-Ac-LDL labelled macrophages were separately embedded in Matrigel drops that were seeded apart from each other with a distance < 500 μm. The cell migration was monitored with Operetta™ for 24 hours.

Video S3 and S4. Representative videos captured with intravital microscopy to show in vivo interactions between hEnSCs and Kupffer cells, hESCs and Kupffer cells
Mouse ALF was induced with intraperitoneal injection of D-GalN 24 hours ahead of cell transplantation. Before cell transplantation, mice were anesthetized with Avertin and a catheter was inserted via tail vein for delivery of the fluorescently labeled antibodies and anesthetic. Surgical preparation for liver intravital imaging was performed as previously described. Kupffer cells in liver sinusoid were visualized by intravenous infusion of PE-conjugated anti-F4/80. 1 x 10^6 EGFP-expressing hEnSCs or H9 hESCs were injected into the portal vein before imaging. Image acquisition was performed using an inverted Olympus FV3000 confocal microscope with a 20x/0.75 UPLANSAPO objective lens. Two fields of interest were acquired simultaneously for each mouse, taken every 90 seconds for 1-3 hours. Data analysis was conducted using ImageJ (FIJI). See details in Methods.
